# Supplementary material for: Immunity against Moraxella catarrhalis requires guanylate‐binding proteins and caspase‐11‐NLRP3 inflammasomes
Source: EMBO J. 2023 Feb 10;42(6):e112558. doi: 10.15252/embj.2022112558 (PMC10015372; doi:10.15252/embj.2022112558)
Supplement: Supplementary file 1 — Appendix [file EMBJ-42-e112558-s007.pdf]

Appendix Data

Cytosolic innate immunity against *Moraxella catarrhalis* requires guanylate-binding proteins and inflammasome activation

Daniel Enosi Tuipulotu[1†], Shouya Feng[1†], Abhimanu Pandey[1], Anyang Zhao[1], Chinh Ngo[1], Anukriti Mathur[1], Jiwon Lee[2], Cheng Shen[1], Daniel Fox[1], Yansong Xue[1], Callum Kay[1], Max Kirkby[1], Jordan Lo Pilato[1], Nadeem O. Kaakoush[3], Daryl Webb[2], Melanie Rug[2], Avril A.B. Robertson[4], Melkamu B. Tessema[5], Stanley Pang[6,7], Daniel Degrandi[8], Klaus Pfeffer[8], Daria Augustyniak[9], Antje Blumenthal[10], Lisa A. Miosge[1], Anne Brüstle[1], Masahiro Yamamoto[11,12], Patrick C Reading[5,13], Gaetan Burgio[1], Si Ming Man[1].

[†] These authors contributed equally: Daniel Enosi Tuipulotu, Shouya Feng

*Correspondence: siming.man@anu.edu.au

Table of Contents

Appendix Figure S1. Innate immune signalling in response to *M. catarrhalis* infection is mediated by TLR4 and cGAS-STING. 2

Appendix Figure S2. Potassium efflux drives inflammasome activation by *M. catarrhalis*...... 3

Appendix Figure S3. *M. catarrhalis* OMVs activate the inflammasome. 5

Appendix Figure S4. *M. catarrhalis* infection induces robust expression of IFN-inducible GTPases. 7

Appendix Figure S5. Generation of *Gbp4/8/9*$^{–/–}$ and *Gbp11*$^{–/–}$ mice.......... 8

Appendix Figure S6. Generation of *Gbp2*$^{–/–}$, *Gbp3*$^{–/–}$ and *Gbp5*$^{–/–}$ mice.......... 9

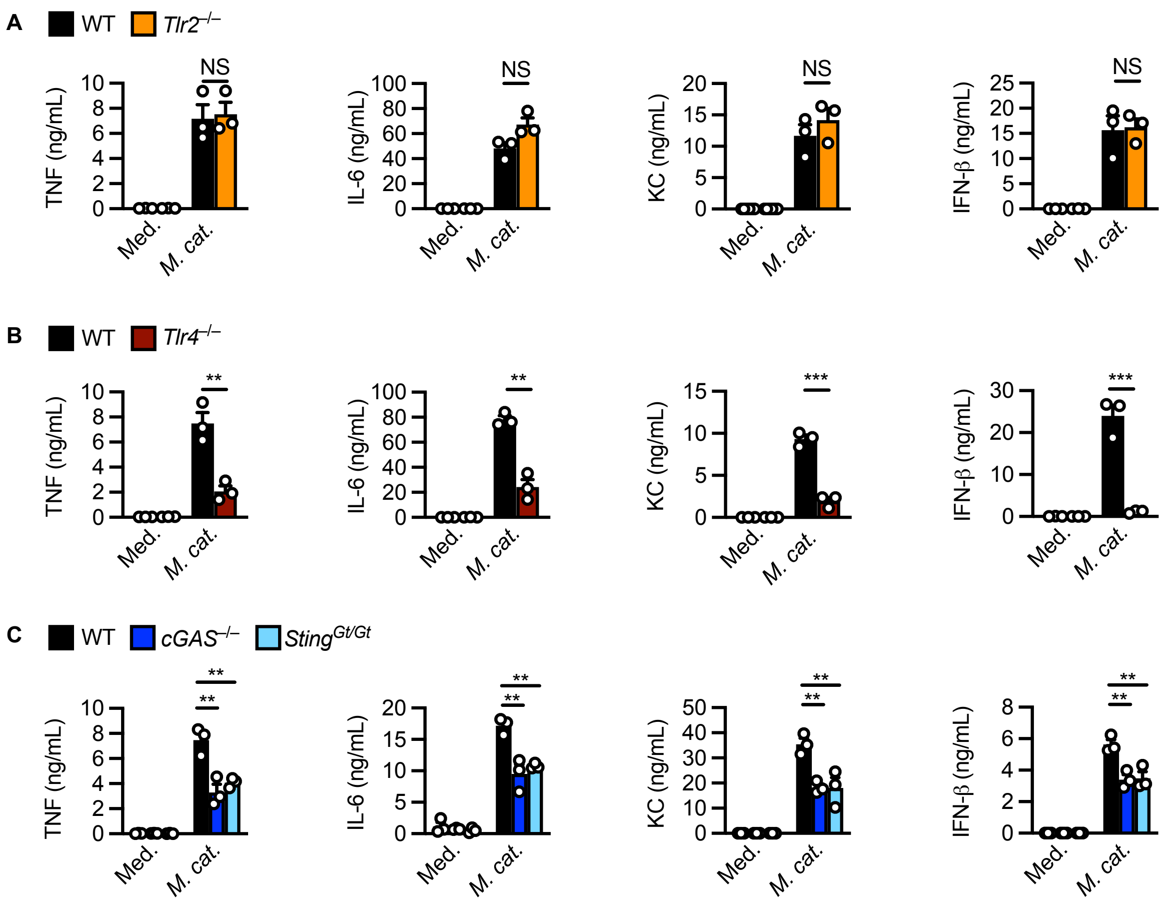

Appendix Figure S1. Innate immune signalling in response to *M. catarrhalis* infection is mediated by TLR4 and cGAS-STING.

A. Release of TNF, IL-6, KC and IFN-β from WT and *Tlr2*$^{-/-}$ BMDMs left untreated (Med.) or assessed 20 h after infection with *M. catarrhalis* (Ne11, MOI 50)

B. Release of TNF, IL-6, KC and IFN-β from WT and *Tlr4*$^{-/-}$ BMDMs left untreated (Med.) or infected as in A.

C. Release of TNF, IL-6, KC and IFN-β from WT, *cGAS*$^{-/-}$ and *Sting*$^{Gt/Gt}$ BMDMs left untreated (Med.) or infected as in A.

Data information: Each symbol represents an independent biological replicate (A-C). NS, no statistical significance; ** P<0.01; *** P<0.001 (two-tailed t-test (A and B) or one-way ANOVA with Dunnett's multiple-comparisons test (C)). Data are pooled from three independent experiments (A-C; mean and s.e.m. in A-C).

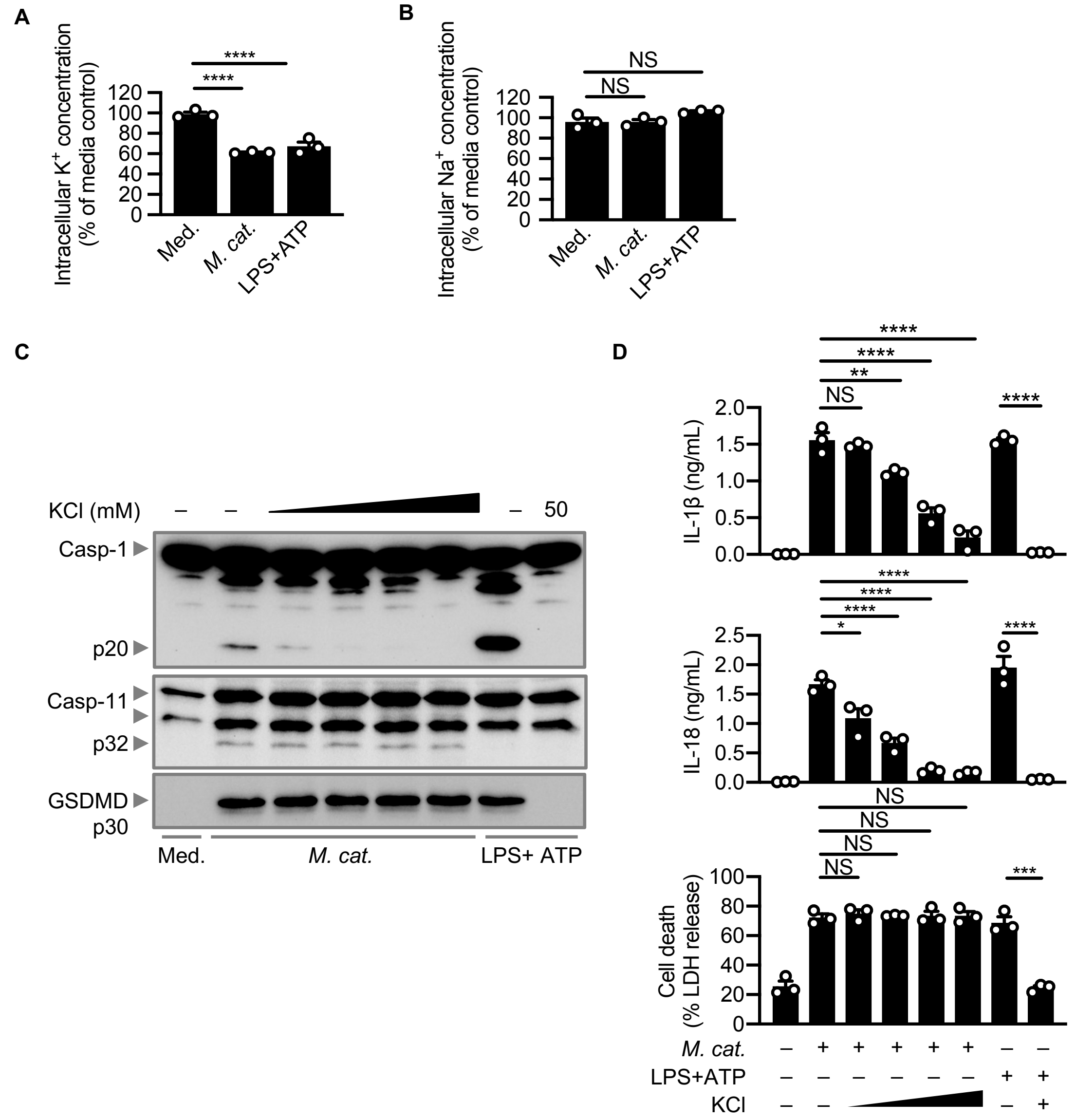

Appendix Figure S2. Potassium efflux drives inflammasome activation by *M. catarrhalis*.

A. Inductively coupled plasma-optical emission spectrometry (ICP-OES) analysis of intracellular K^+ concentration of WT BMDMs left untreated (Med.), assessed 16 h after infection with *M. catarrhalis* (Ne11, MOI 100), or 30 min after treatment with LPS+ATP (5 mM).

B. ICP-OES analysis of intracellular Na^+ of WT BMDMs treated as in A.

C. Immunoblot analysis of caspase-1 (Casp-1), capase-11 (Casp-11) and gasdermin D (GSDMD) in WT BMDMs left untreated (Med.) or assessed 10 h after infection with *M. catarrhalis* (Ne11, MOI 100) in the absence (-) or presence of KCl at 50 mM, or in the presence of increasing concentration of KCl (wedge; 10, 25, 50 and 75 mM).

D. Release of IL-1β, IL-18 and LDH from BMDMs after treatment as in C.

Data information: Each symbol represents an independent biological replicate (A, B, D). NS, no statistical significance; * $P<0.05$; ** $P<0.01$; *** $P<0.001$; **** $P<0.0001$ (one-way ANOVA (A, B, D)). Data are from one experiment, representative of three independent experiments (C) or are pooled from three independent experiments (A, B, D; mean and s.e.m. in A, B, D).

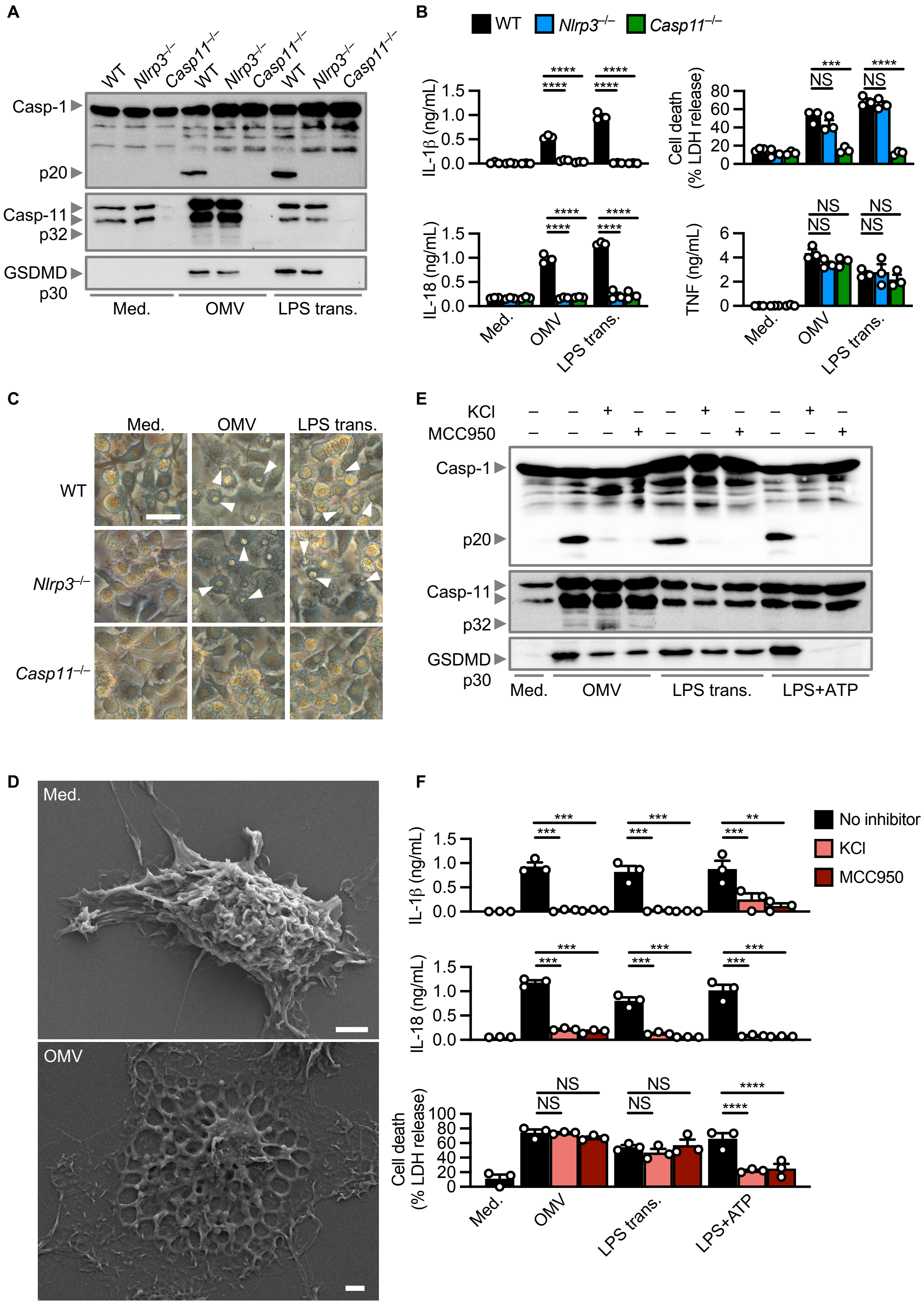
A
WT
Nlrp3–/–
Casp11–/–
Casp-1
p20
Casp-11
p32
GSDMD
p30
Med.
OMV
LPS trans.
B
WT
Nlrp3–/–
Casp11–/–
IL-1β (ng/mL)
Cell death (% LDH release)
IL-18 (ng/mL)
TNF (ng/mL)
NS
Med.
OMV
LPS trans.
C
Med.
OMV
LPS trans.
WT
Nlrp3–/–
Casp11–/–
D
Med.
OMV
E
KCl
MCC950
Casp-1
p20
Casp-11
p32
GSDMD
p30
Med.
OMV
LPS trans.
LPS+ATP
F
No inhibitor
KCl
MCC950
IL-1β (ng/mL)
IL-18 (ng/mL)
Cell death (% LDH release)
NS
Med.
OMV
LPS trans.
LPS+ATP

Appendix Figure S3. *M. catarrhalis* OMVs activate the inflammasome.

A. Immunoblot analysis of caspase-1 (Casp-1), caspase-11 (Casp-11) and gasdermin D (GSDMD) in WT, *Nlrp3*$^{-/-}$ and *Casp11*$^{-/-}$ BMDMs left untreated (Med.) or assessed 20 h after incubation with 10 µg of OMVs purified from *M. catarrhalis* (Ne11), or 5 h after transfection with 5 µg of *E. coli* LPS.

B. Release of IL-1β, IL-18, TNF and LDH from BMDMs after treatment as in A.

C. Brightfield microscopy analysis of WT and mutant BMDMs after treatment as in A.

D. Scanning electron microscopy of WT BMDMs left untreated (Med.) or assessed 20 h after incubation with 10 µg of OMVs purified from *M. catarrhalis* (Ne11).

E. Immunoblot analysis of Casp-1, Casp-11 and GSDMD in WT BMDMs left untreated (Med.) or assessed 20 h after incubation with 10 µg of OMVs purified from *M. catarrhalis* (Ne11), or 5 h after transfection with 5 µg of *E. coli* LPS, or 30 min after treatment with LPS+ATP (5 mM) in the absence (-) or presence (+) of 50 mM KCl (+) or 20 µM of the NLRP3 inhibitor MCC950 (+).

F. Release of IL-1β, IL-18 and LDH from BMDMs after treatment as in E.

Data information: Arrowheads indicate dead cells. Each symbol represents an independent biological replicate (B and F). NS, no statistical significance; ** $P<0.01$; *** $P<0.001$; **** $P<0.0001$ (one-way ANOVA with Dunnett's multiple-comparisons test (B and F)). Data are from one experiment representative of two (D) or three independent experiments (A, C, E) or are pooled from three independent experiments (B and F; mean and s.e.m. in B and F). Scale bars, 20 µm (C), 2 µm (D).

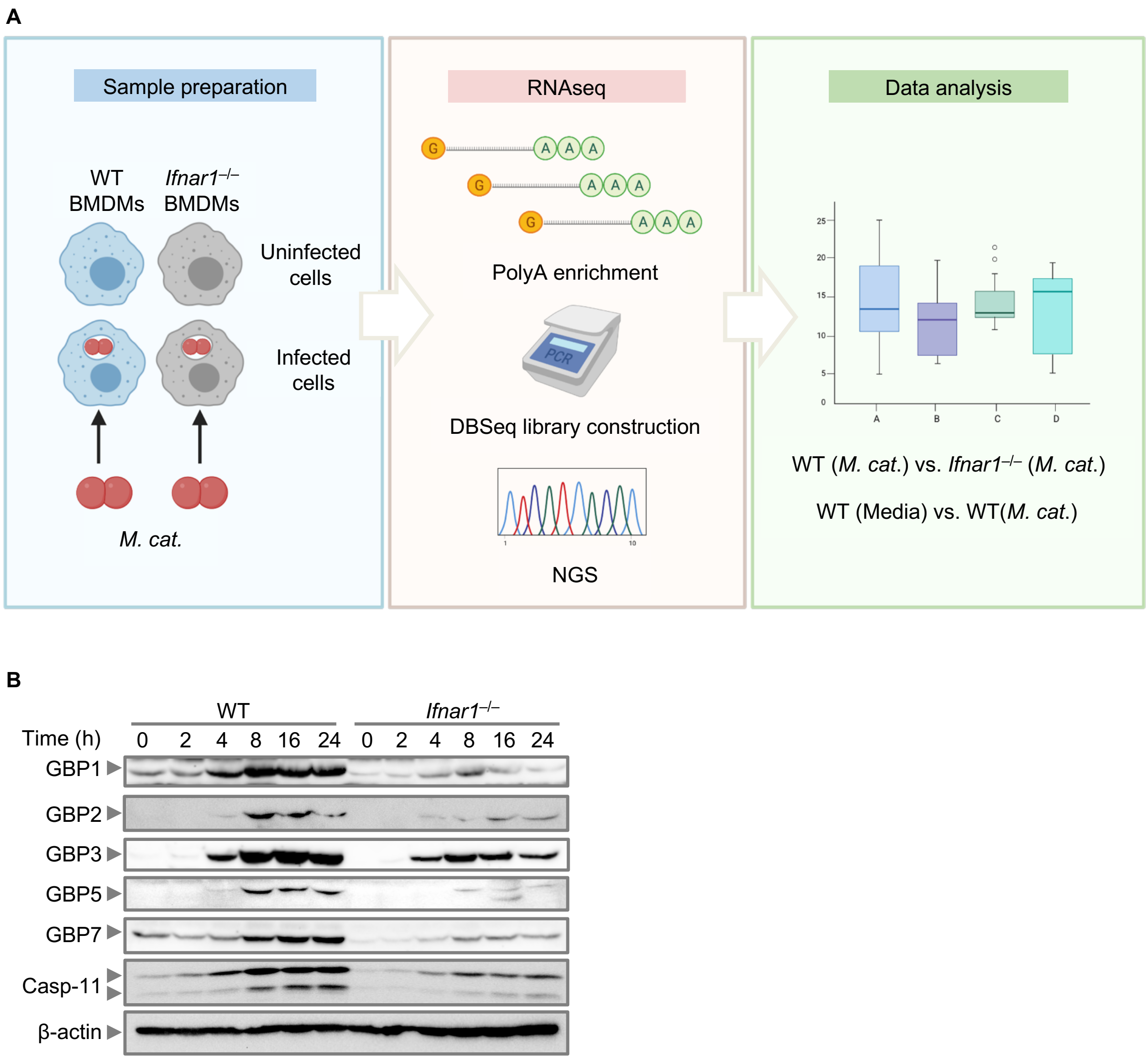

Appendix Figure S4. ***M. catarrhalis*** **infection induces robust expression of IFN-inducible GTPases.**

A. Schematic overview of RNA-sequencing experiments, including the experimental conditions, sample preparation and bioinformatic analyses.

B. Immunoblot analysis of GBP1, GBP2, GBP3, GBP5, GBP7, caspase-11 (Casp-11) and β-actin in WT and *Ifnar1*$^{-/-}$ BMDMs 0-24 h after infection with *M. catarrhalis* (Ne11, MOI 50).

Data information: Data are from one experiment representative of three independent experiments (B).

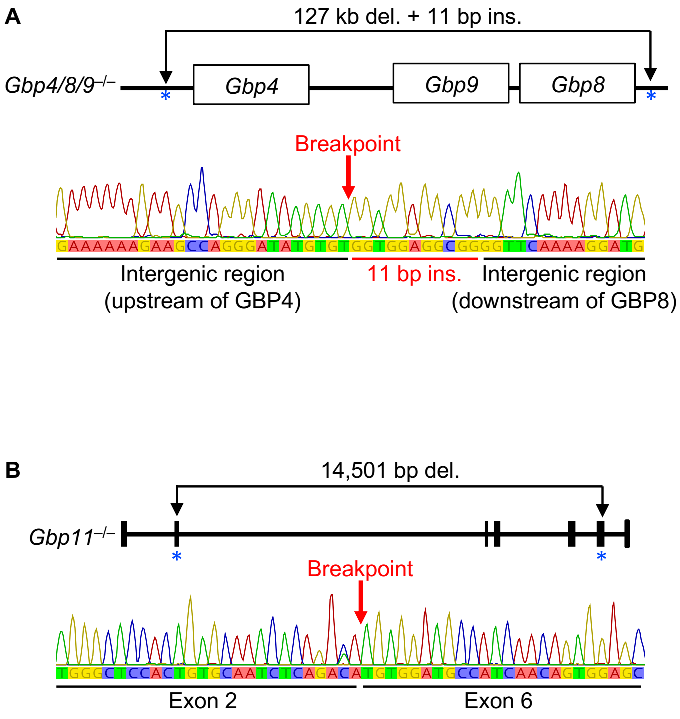

Appendix Figure S5. Generation of *Gbp4/8/9*$^{-/-}$ and *Gbp11*$^{-/-}$ mice.

A. Schematic of the sgRNA-targeted intergenic regions upstream of *Gbp4* and downstream of *Gbp8* in the *Gbp4/8/9*$^{-/-}$ mouse strain and Sanger sequencing across the breakpoint.

B. Schematic of the sgRNA-targeted exons of *Gbp11* in the *Gbp11*$^{-/-}$ mouse strain and Sanger sequencing across the breakpoint.

Data information: Blue asterisks (*) indicate gRNA targets (exons or intergenomic regions), “del.” indicates deletion, and “ins.” indicates insertion.

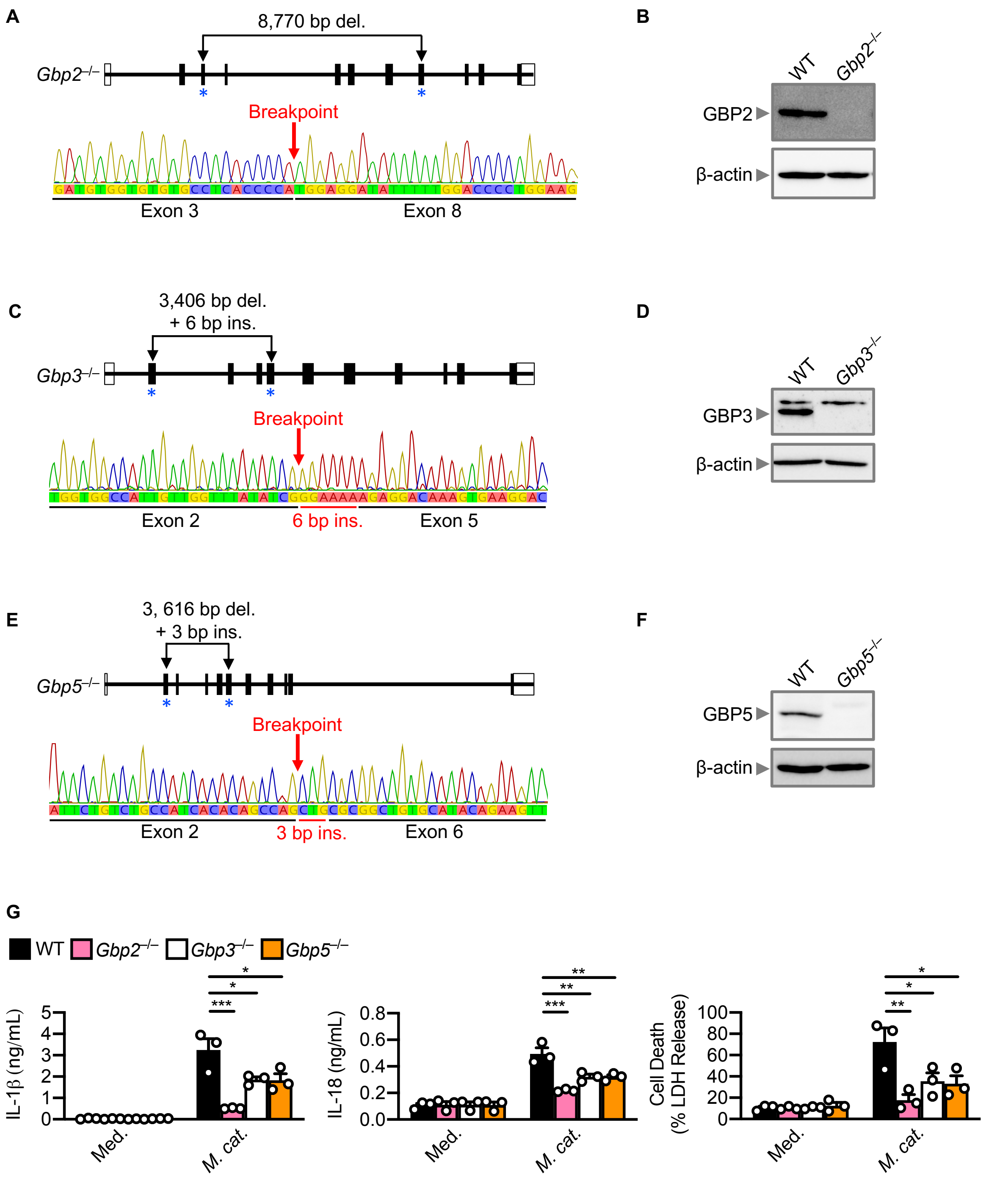

Appendix Figure S6. Generation of *Gbp2*$^{-/-}$, *Gbp3*$^{-/-}$ and *Gbp5*$^{-/-}$ mice.

A. Schematic of the sgRNA-targeted exons of *Gbp2* in the *Gbp2*$^{-/-}$ mouse strain and Sanger sequencing across the breakpoint.

B. Immunoblot analysis of GBP2 (top) and β-actin (bottom) in WT and *Gbp2*$^{-/-}$ colon tissues.

C. Schematic of the sgRNA-targeted exons of *Gbp3* in the *Gbp3*$^{-/-}$ mouse strain and Sanger sequencing across the breakpoint.

D. Immunoblot analysis of GBP3 (top) and β-actin (bottom) in WT and *Gbp3*$^{-/-}$ colon tissues.

E. Schematic of the sgRNA-targeted exons of *Gbp5* in the *Gbp5*$^{-/-}$ mouse strain and Sanger sequencing across the breakpoint.

F. Immunoblot analysis of GBP5 (top) and β-actin (bottom) in WT and *Gbp5*$^{-/-}$ colon tissues.

G. Release of IL-1β, IL-18 and LDH from WT, *Gbp2*$^{-/-}$ *Gbp3*$^{-/-}$ and *Gbp5*$^{-/-}$ BMDMs left untreated (Med.) or assessed 10 h after infection with *M. catarrhalis* (Ne11, MOI 100).

Data information: Each symbol represents an independent biological replicate (G). ** P<0.01; *** P<0.001 (one-way ANOVA with Dunnett's multiple-comparisons test (G)). Data are representative of two (B, D, F) or three independent experiments (G; mean and s.e.m. in G). Blue asterisks (*) indicate gRNA targets (exons), "del." indicates deletion, and "ins." indicates insertion.
