# Supplementary material for: Immunity against Moraxella catarrhalis requires guanylate‐binding proteins and caspase‐11‐NLRP3 inflammasomes
Source: EMBO J. 2023 Feb 10;42(6):e112558. doi: 10.15252/embj.2022112558 (PMC10015372; doi:10.15252/embj.2022112558)
Supplement: Supplementary file 2 — Expanded View Figures PDF [file EMBJ-42-e112558-s009.pdf]

Expanded View Figures

Figure EV1. Global innate responses are unaltered in *Casp11*$^{-/-}$ BMDMs during *Moraxella catarrhalis* infection compared with WT BMDMs.

A Brightfield microscopy analysis of WT and mutant BMDMs left untreated (Med.) or assessed 20 h after infection with *M. catarrhalis* (Ne11, MOI 100).
B Immunoblot analysis of phospho-IkB (pIkB), IkB, phospho-ERK (pERK), ERK, caspase-11 (Casp-11) and GAPDH (loading control) of WT and *Casp11*$^{-/-}$ BMDMs 0–60 min after infection with *M. catarrhalis* (Ne11 strain, MOI 50).
C Immunoblot analysis of caspase-1 (Casp-1), Casp-11 and gasdermin D (GSDMD) in WT BMDMs left untreated (Med.) or assessed 20 h after infection with *M. catarrhalis* (Ne11, MOI 100), 30 min after treatment with LPS + ATP (5 mM) or 5 h after transfection with 5 μg of *Escherichia coli* LPS in the absence (–) or presence (+) of the NLRP3 inhibitor MCC950 (20 μM).
D Release of IL-1β, IL-18 and LDH from BMDMs after treatment as in (C).
E Release of KC and IL-6 in WT and *Casp11*$^{-/-}$ BMDMs left untreated (Med.) or infected as in (A).
F qRT–PCR analysis of the genes encoding Casp-11, KC (*Cxcl1*), IL-1β, IL-18, IL-6 and TNF in WT and *Casp11*$^{-/-}$ BMDMs left untreated or assessed 4 h after infection with *M. catarrhalis* (Ne11 strain, MOI 50), relative to *Gapdh*.

Data information: Arrowheads indicate dead cells (A). Each symbol represents an independent biological replicate (D–F). NS, no statistical significance; $**P < 0.01$; $***P < 0.001$; $****P < 0.0001$ (two-tailed *t*-test (D–F)). Data are from one experiment representative of three independent experiments (A–C) or are pooled from three independent experiments (D–F; mean and s.e.m. in D–F). Scale bar, 20 μm (A).

© 2023 The Authors

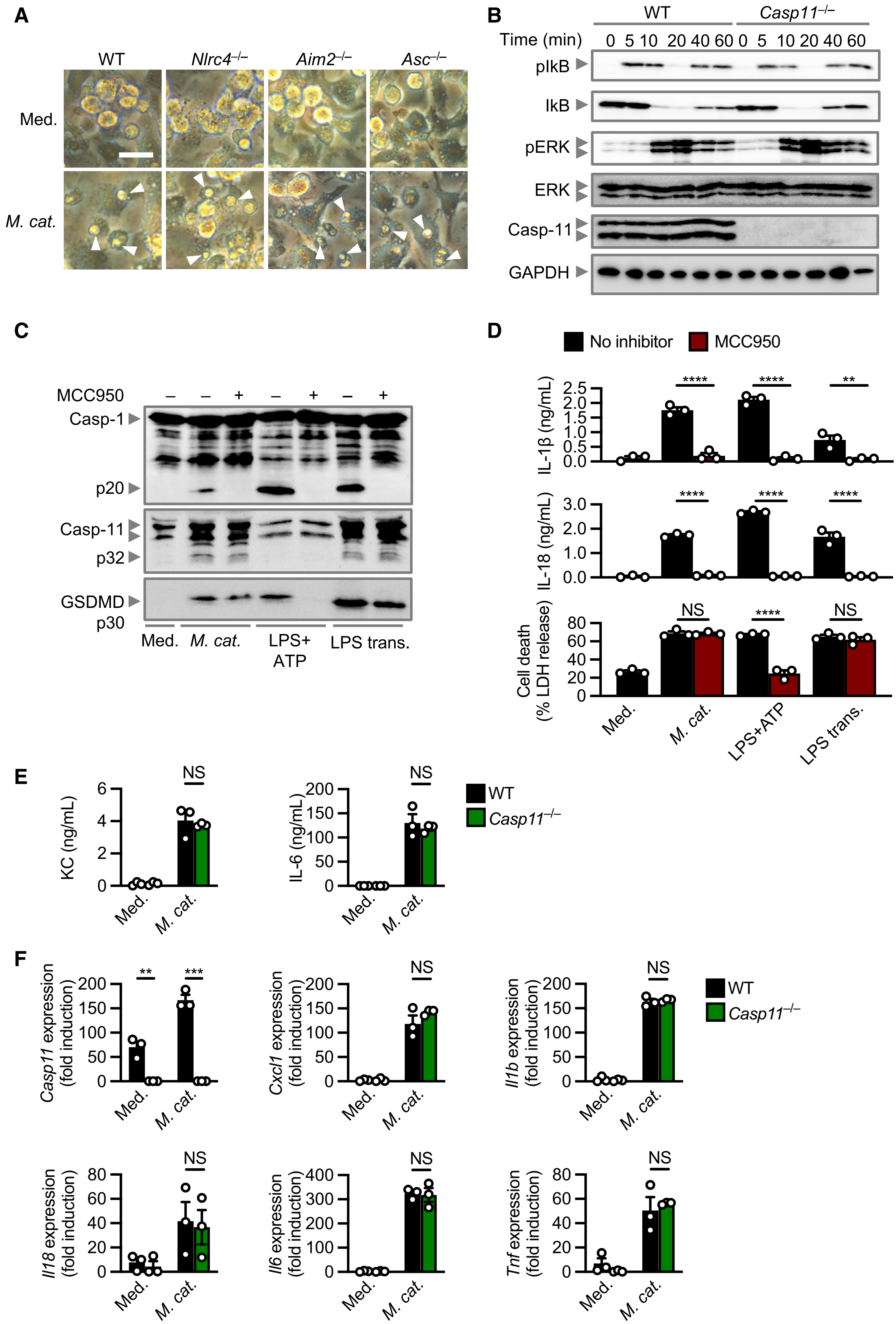

Figure EV1.

 © 2023 The Authors

Figure EV2. Activation of the caspase-4/11 inflammasome is conserved amongst strains of *Moraxella catarrhalis*.

A Immunoblot analysis of caspase-1 (Casp-1), caspase-11 (Casp-11) and gasdermin D (GSDMD) in WT and *Casp11*$^{-/-}$ BMDMs left untreated (Med.) or assessed 10 h after infection with 12 strains of *M. catarrhalis* (MOI 50), or 30 min after treatment with LPS + ATP (5 mM).

B Release of IL-1β, IL-18, TNF and LDH from BMDMs after treatment as in (A).

C Release of IL-1β, IL-18 and LDH from WT and *Casp4*$^{-/-}$ THP-1 macrophage-like cells left untreated (Med.), assessed 6 h after infection with *M. catarrhalis* (Ne11, MOI 50), following overnight transfection with 5 μg of *Escherichia coli* LPS or after Pam3CSK4 priming with overnight transfection with 5 μg of poly(dA:dT).

D IncuCyte live-imaging analysis of WT and *Casp4*$^{-/-}$ THP-1 macrophage-like cell viability assessed after treatment as in (C).

Data information: Each symbol represents an independent biological replicate (B and C). NS, no statistical significance; *$P < 0.05$; **$P < 0.01$; ***$P < 0.001$; ****$P < 0.0001$ (two-tailed *t*-test (B–D)). Data are from one experiment representative three independent experiments (A) or are pooled from three independent experiments (B–D; mean and s.e.m. in B–D).

© 2023 The Authors

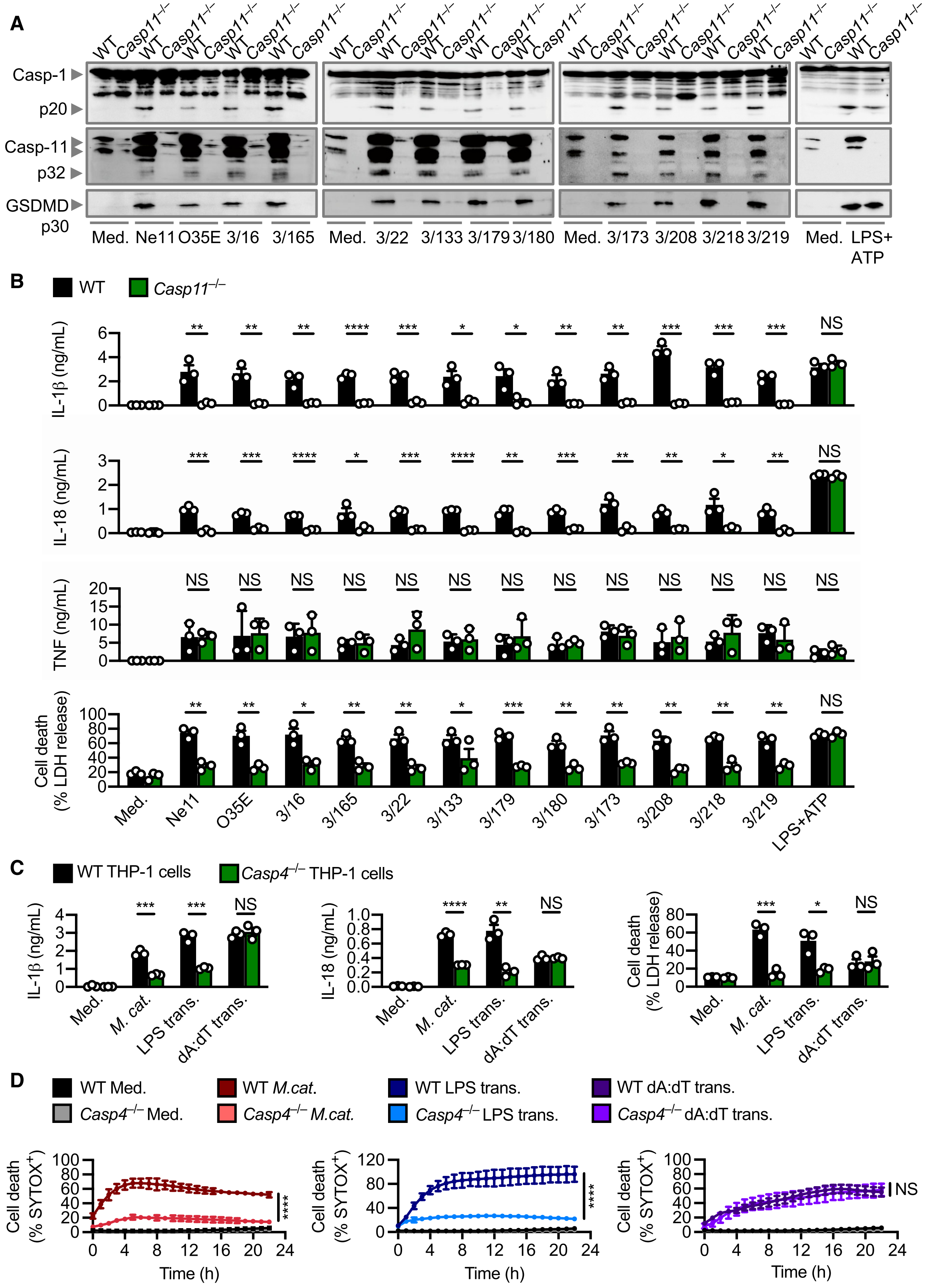

Figure EV2.

 © 2023 The Authors

Figure EV3. ***Moraxella catarrhalis*** **lacking LOS cannot activate the inflammasome.**

A–D Scanning electron microscopy (SEM) of *M. catarrhalis* O35E and Δ*lpxA*.
E Confocal microscopy analysis of intracellular *M. catarrhalis* (green) in WT BMDMs 12 h after infection with O35E or Δ*lpxA* (MOI 20).
F Brightfield microscopy analysis of WT, *Nlrp3*$^{-/-}$ and *Casp11*$^{-/-}$ BMDMs left untreated (Med.) or assessed 10 h after infection with *M. catarrhalis* (O35E and Δ*lpxA*, MOI 100).
G Immunoblot analysis of caspase-1 (Casp-1) and gasdermin D (GSDMD) in WT BMDMs left untreated (Med.) or assessed 10 h after infection with O35E or Δ*lpxA* (MOI 100) either in the absence of priming or following 3 h of priming with Pam3CSK4 (0.5 μg/ml) or IFN-γ (100 U/ml).
H Release of IL-1β, IL-18, TNF and LDH from WT BMDMs after treatment as in (G).
I Brightfield microscopy analysis of WT BMDMs after treatment as in (G).

Data information: Arrowheads indicate dead cells (F and I). Each symbol represents an independent biological replicate (H). NS, no statistical significance; ****$P < 0.0001$ (one-way ANOVA with Dunnett's multiple comparisons test (H)). Data are from one experiment representative of two (A–E) or three independent experiments (F, G, I) or are pooled from three independent experiments (H; mean and s.e.m. in H). Scale bars, 1 μm (A and B), 200 nm (C and D), 5 μm (E), 20 μm (F and I).

© 2023 The Authors

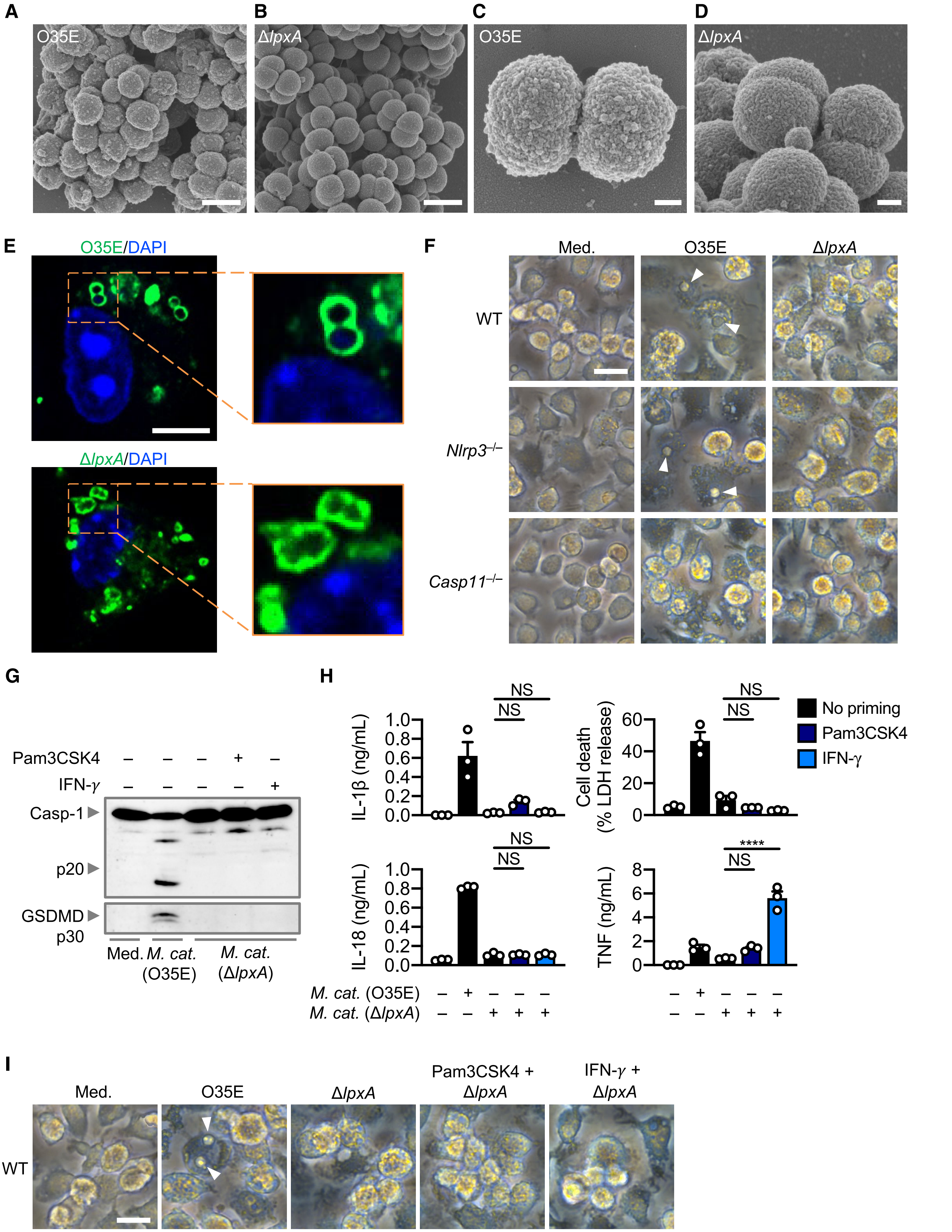

Figure EV3.

 © 2023 The Authors

Figure EV4. Recruitment of GBPs to intracellular *Moraxella catarrhalis* is not affected by the absence of LOS.

A Immunoblot analysis of caspase-1 (Casp-1), caspase-11 (Casp-11) and gasdermin D (GSDMD) in WT, *Gbp*^{Chr3}-KO and *Casp11*$^{-/-}$ BMDMs left untreated (Med.) or 10 h after transfection with 5 μg of LPS from *Escherichia coli* or 5 μg of LOS from *M. catarrhalis* (O35E).

B Release of IL-1β, IL-18, TNF and LDH from BMDMs after treatment as in (A).

C Confocal microscopy analysis of FLAG-OVA, FLAG-GBP1, FLAG-GBP2, FLAG-GBP3, FLAG-GBP5 (red) and *M. catarrhalis* (green) in LA-4 cells left untreated (Med.), or 16 h after infection of IFN-γ-primed (100 U/ml) LA-4 cells with *M. catarrhalis* (O35E or Δ*lpxA*, MOI 20).

D Quantitation of FLAG-OVA, FLAG-GBP1, FLAG-GBP2, FLAG-GBP3 and FLAG-GBP5 positive *M. catarrhalis* in LA-4 cells treated as in (C).

Data information: Arrowheads indicate bacteria colocalised with GBPs (C). Each symbol represents an independent biological replicate (B). NS, no statistical significance (one-way ANOVA with Dunnett's multiple-comparisons test (B and D)). To quantify the bacterial number in a field of view, we used the single green channel (anti-*M. catarrhalis* staining), where a single bacterium is defined as an intact circle. To quantify the proportion of GBP-positive bacteria in a field of view, we used the single red channel (anti-GBP staining) and compared this to the green channel (anti-*M. catarrhalis* staining). The number of GBP-positive *M. catarrhalis* was divided by the total number of *M. catarrhalis* counted to obtain the proportion of GBP-positive bacteria. A total of 100 lung epithelial cells (LA-4) were analysed to quantify the proportion of GBP-positive bacteria per cell using confocal microscopy (D). Data are from one experiment representative of two (C) or three independent experiments (A) or are pooled from two (D) or three independent experiments (B; mean and s.e.m. in B and D). Scale bars, 4 μm (C).

© 2023 The Authors

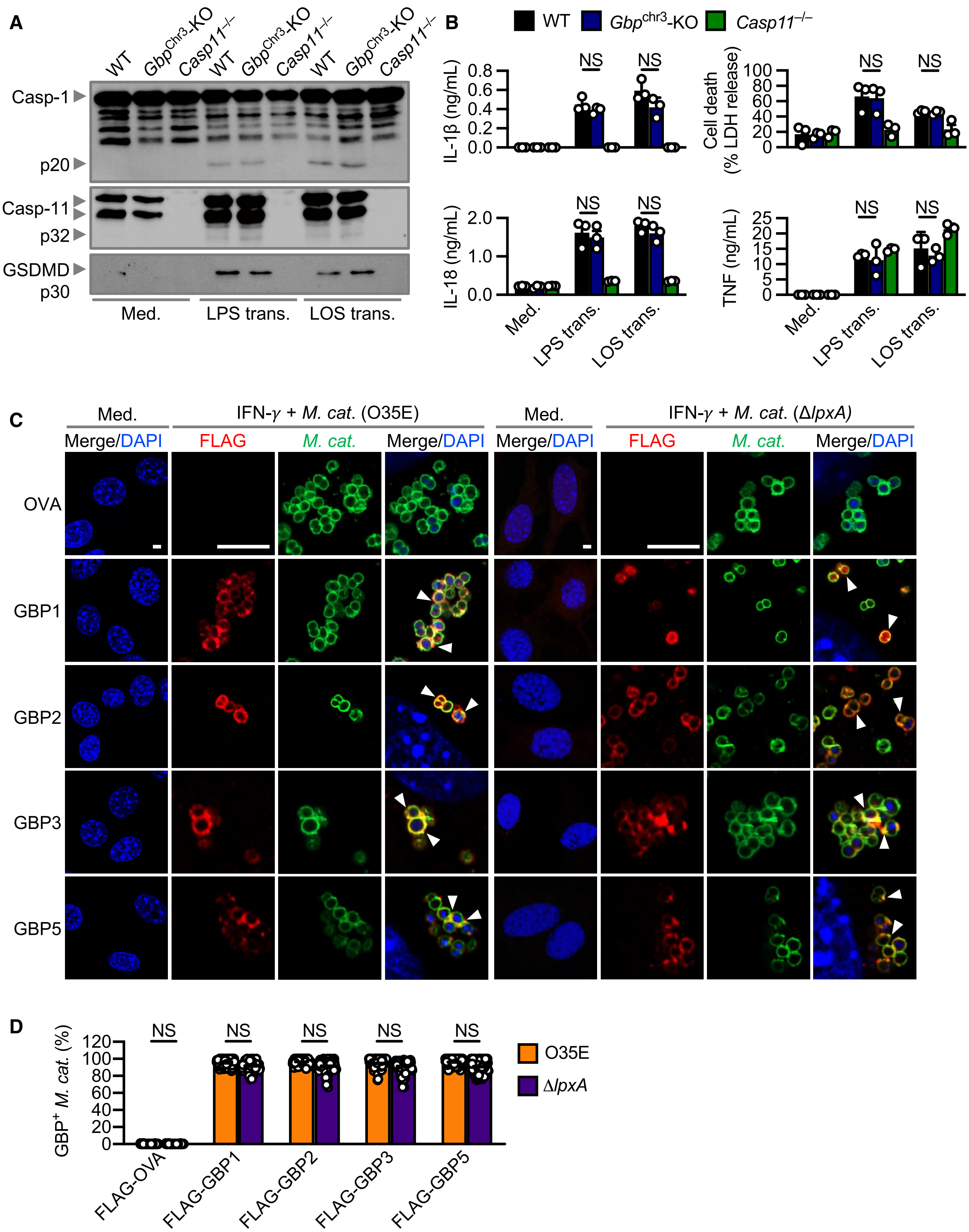

Figure EV4.

 © 2023 The Authors

Figure EV5. GBPs mediate inflammasome activation by *Moraxella catarrhalis* OMVs.

A Immunoblot analysis of caspase-1 (Casp-1), caspase-11 (Casp-11) and gasdermin D (GSDMD) in WT, *Gbp*^{chr3}-KO and *Ifnar1*$^{-/-}$ BMDMs left untreated (Med.) or primed with IFN-γ (100 U/ml) and assessed 10 h after incubation with 10 μg of *M. catarrhalis* OMVs (Ne11).

B Release of IL-1β, IL-18, TNF and LDH from BMDMs after treatment as in (A).

C Immunoblot analysis of Casp-1, Casp-11 and GSDMD in WT, *Gbp1*$^{-/-}$, *Gbp2*$^{-/-}$, *Gbp3*$^{-/-}$, *Gbp5*$^{-/-}$, *Gbp7*$^{-/-}$, and *Ifnar1*$^{-/-}$ BMDMs left untreated (Med.) or treated as in (A).

D Release of IL-1β, IL-18, TNF and LDH from BMDMs after treatment as in (C).

E Immunoblot analysis of Casp-1, Casp-11 and GSDMD in WT, *Gbp4/8/9*$^{-/-}$, *Gbp11*$^{-/-}$ and *Ifnar1*$^{-/-}$ BMDMs left untreated (Med.) or treated as in (A).

F Release of IL-1β, IL-18, TNF and LDH from BMDMs after treatment as in (E).

Data information: Each symbol represents an independent biological replicate (B, D, F). NS, no statistical significance; $^{**}P < 0.01$; $^{***}P < 0.001$; $^{****}P < 0.0001$ (one-way ANOVA with Dunnett's multiple-comparisons test (B, D, F)). Data are from one experiment representative of three independent experiments (A, C, E) or are pooled from three independent experiments (B, D, F; mean and s.e.m. in B, D, F).

© 2023 The Authors

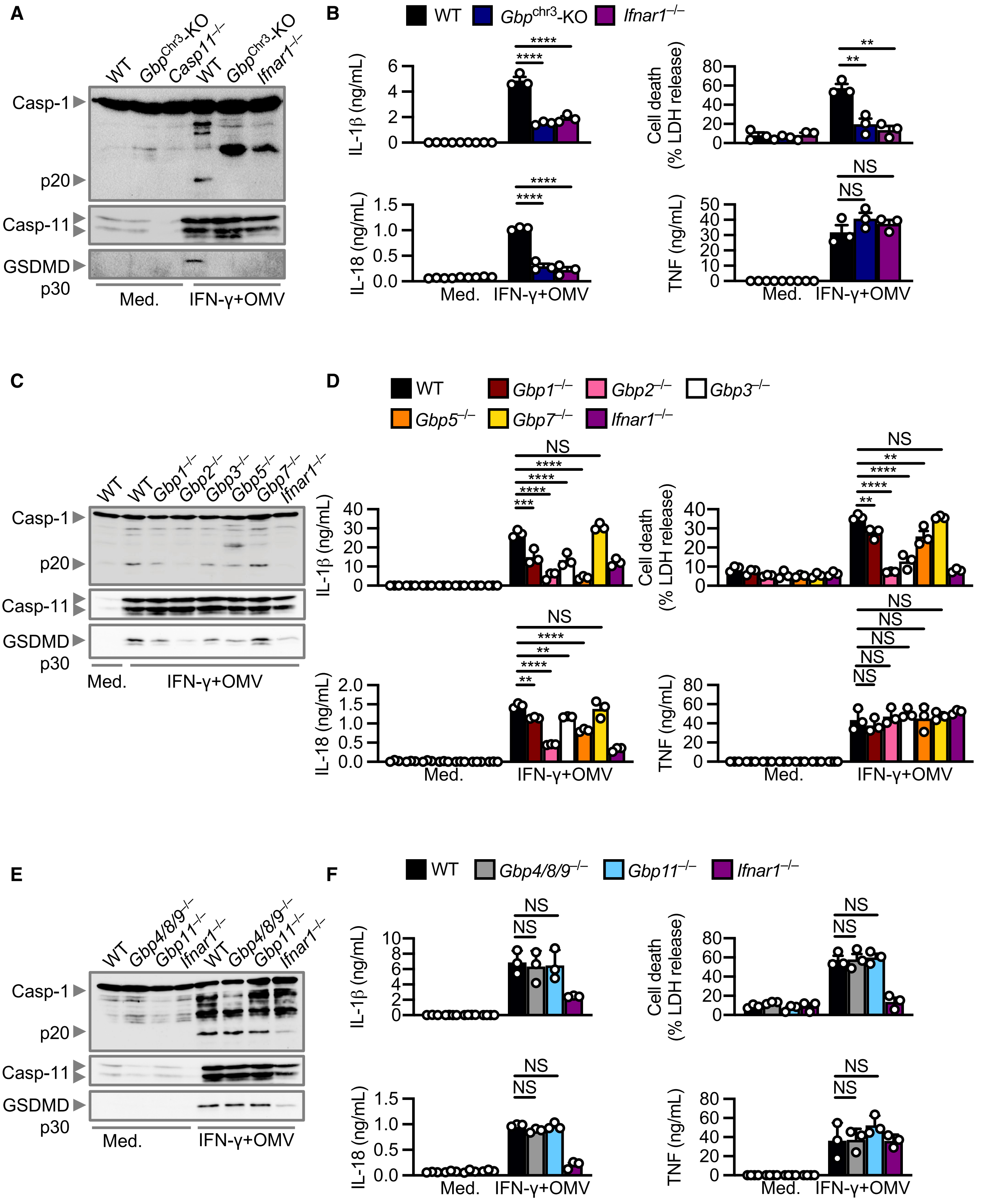

Figure EV5.

© 2023 The Authors
