# Supplementary material for: Immunity against Moraxella catarrhalis requires guanylate‐binding proteins and caspase‐11‐NLRP3 inflammasomes
Source: EMBO J. 2023 Feb 10;42(6):e112558. doi: 10.15252/embj.2022112558 (PMC10015372; doi:10.15252/embj.2022112558)

Figure 1D

- WT, *Nlrp3*$^{-/-}$, *Nlrc4*$^{-/-}$, *Aim2*$^{-/-}$, *Asc*$^{-/-}$, *Casp1/11*$^{-/-}$, *Casp11*$^{-/-}$, *Gsdmd*$^{I105N/I105N}$ BMDMs
- Media, *M. catarrhalis* infection

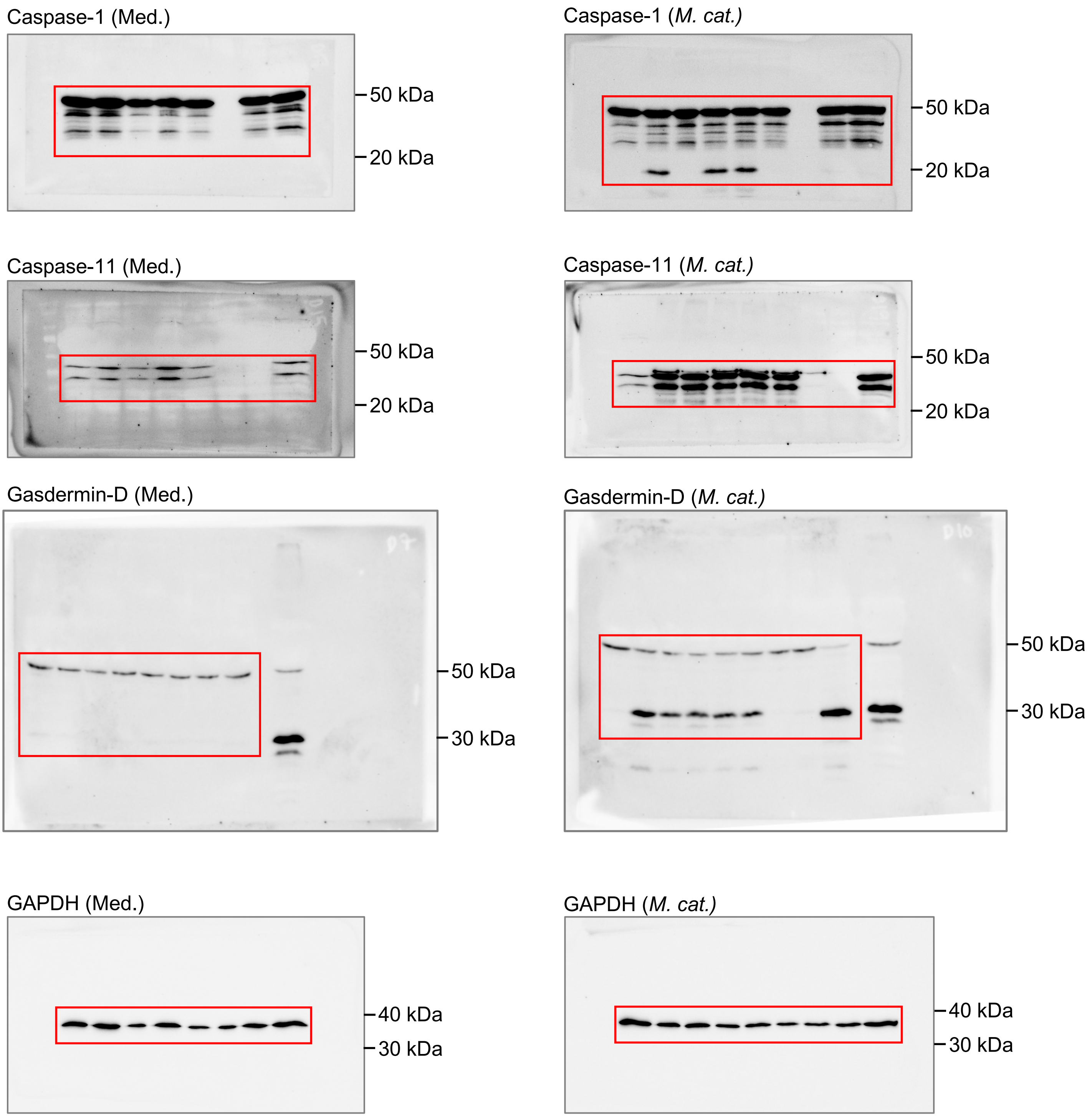

Supplement: Supplementary file 5 — Source Data for Figure 1 [file EMBJ-42-e112558-s003.zip › EMBOJ2022112558_SourceDataForFigure1(D,F,H,I)/D/Western Blots.pdf]
