# Supplementary figures and images for: Immunity against Moraxella catarrhalis requires guanylate‐binding proteins and caspase‐11‐NLRP3 inflammasomes
Source: EMBO J. 2023 Feb 10;42(6):e112558. doi: 10.15252/embj.2022112558 (PMC10015372; doi:10.15252/embj.2022112558)

Figure 1F

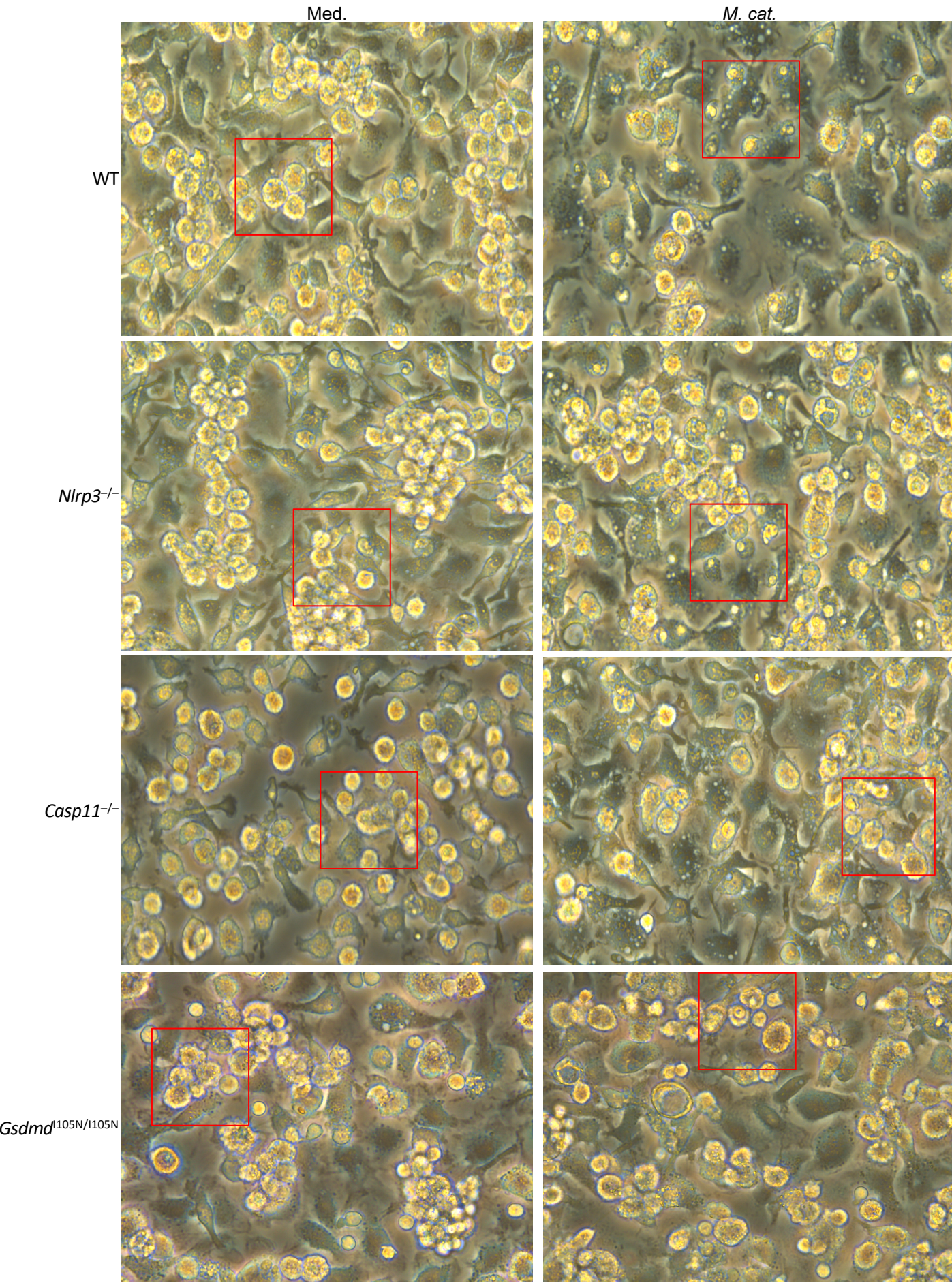

Supplement: Supplementary file 5 — Source Data for Figure 1 [file EMBJ-42-e112558-s003.zip › EMBOJ2022112558_SourceDataForFigure1(D,F,H,I)/F/Microscopy Image Bright-Field.pdf]

Figure 1H

Med.

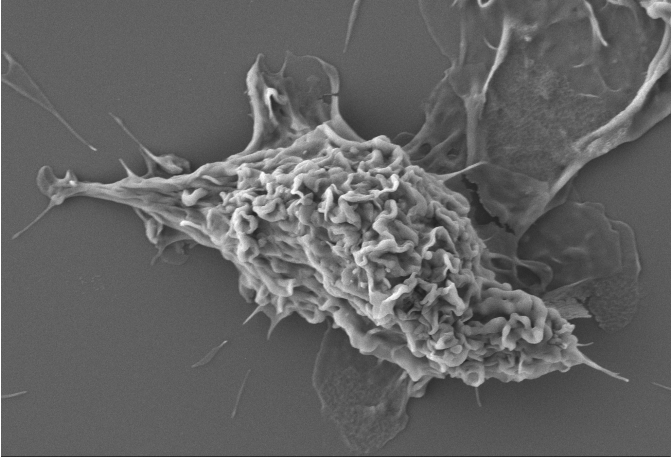

*M. cat.*

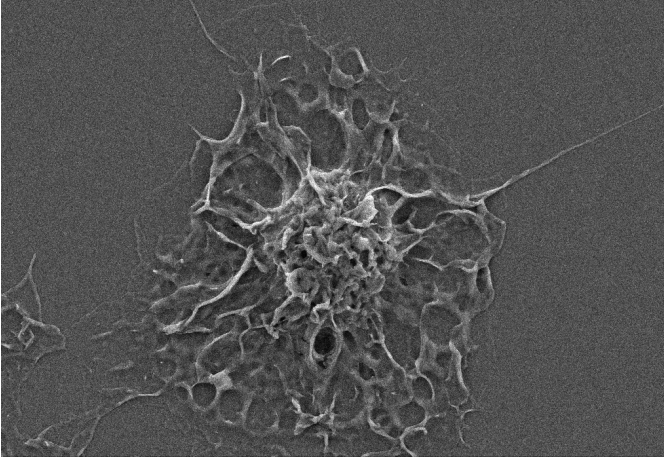

Supplement: Supplementary file 5 — Source Data for Figure 1 [file EMBJ-42-e112558-s003.zip › EMBOJ2022112558_SourceDataForFigure1(D,F,H,I)/H/Microscopy Image EM.pdf]

Figure 11

Med.

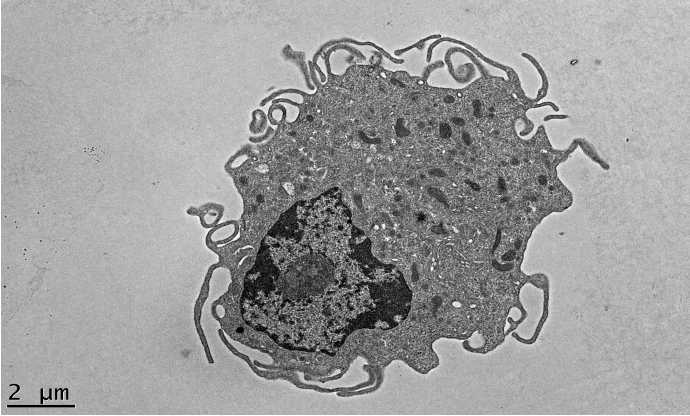

*M. cat.*

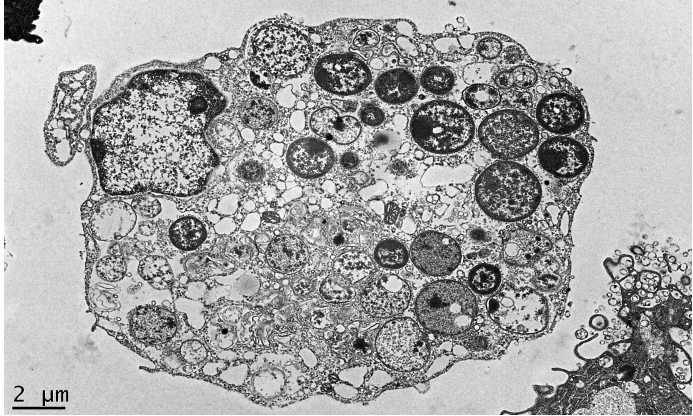

Supplement: Supplementary file 5 — Source Data for Figure 1 [file EMBJ-42-e112558-s003.zip › EMBOJ2022112558_SourceDataForFigure1(D,F,H,I)/I/Microscopy Image EM.pdf]

## Figure 2G

➤ *M. catarrhalis* (O35E) LOS purification, *M. catarrhalis* ( $\Delta$ *pxA*) LOS purification

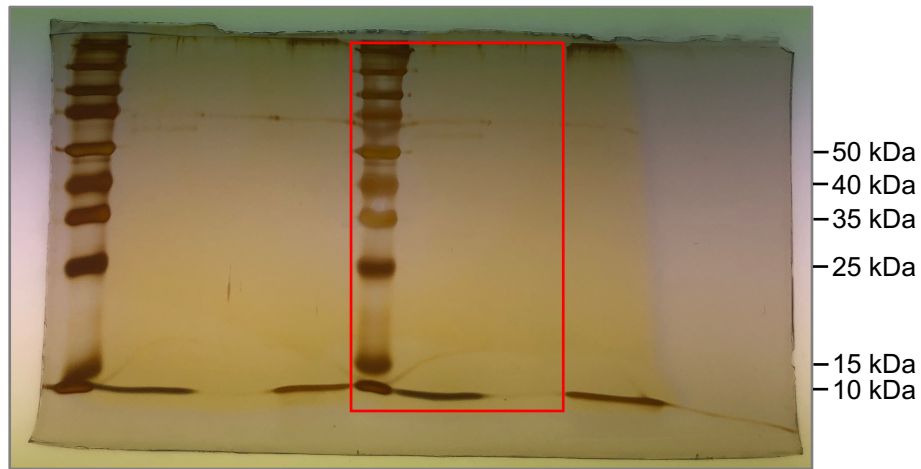

Supplement: Supplementary file 6 — Source Data for Figure 2 [file EMBJ-42-e112558-s002.zip › EMBOJ2022112558_SourceDataForFigure2(A,C,G,H,I)/G/Coomassie Gel.pdf]

Figure 2H

OMV (O35E)

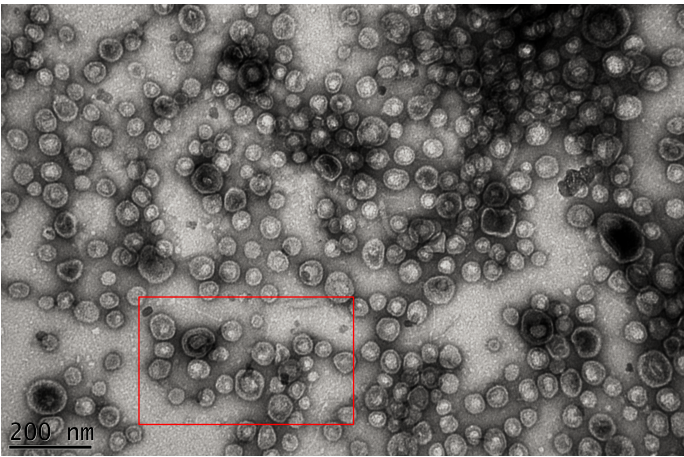

Supplement: Supplementary file 6 — Source Data for Figure 2 [file EMBJ-42-e112558-s002.zip › EMBOJ2022112558_SourceDataForFigure2(A,C,G,H,I)/H/Microscopy Image EM.pdf]

Figure 2I

OMV ( $\Delta$ *pxA*)

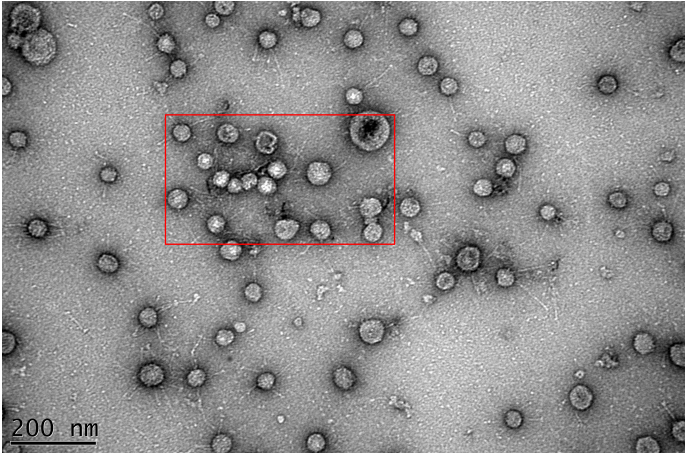

Supplement: Supplementary file 6 — Source Data for Figure 2 [file EMBJ-42-e112558-s002.zip › EMBOJ2022112558_SourceDataForFigure2(A,C,G,H,I)/I/Microscopy Image EM.pdf]

**Figure 3A**  
➤ WT, *Ifnar1*<sup>-/-</sup> BMDMs  
➤ Media, *M. catarrhalis* infection

Caspase-1

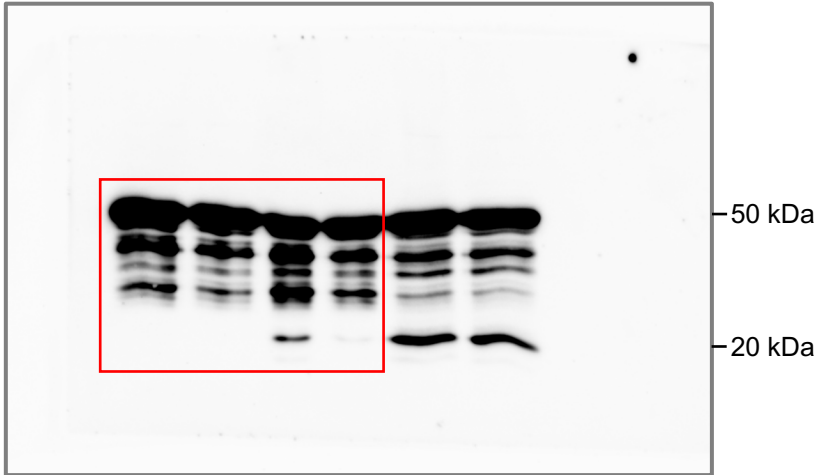

Caspase-11

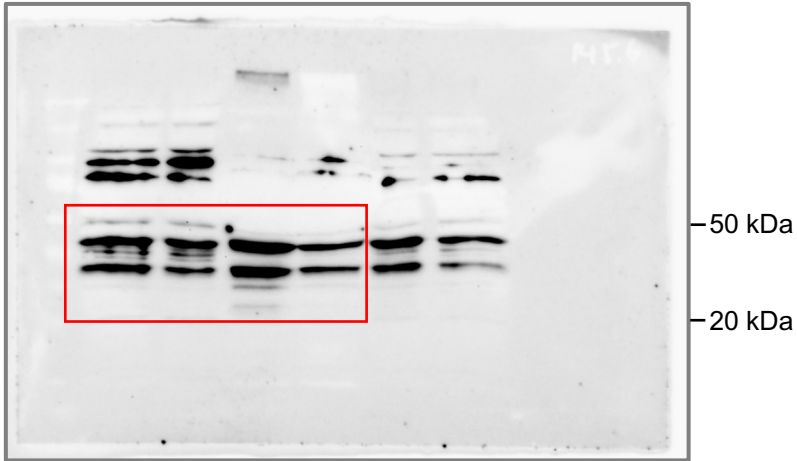

Gasdermin-D

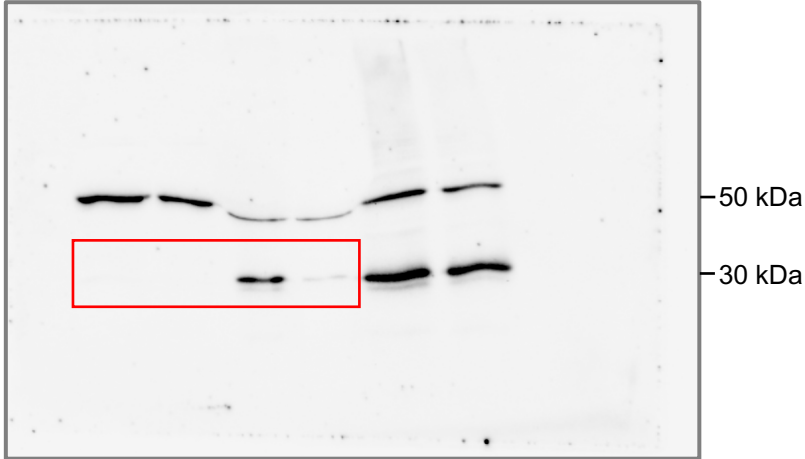

Supplement: Supplementary file 7 — Source Data for Figure 3 [file EMBJ-42-e112558-s008.zip › EMBOJ2022112558_SourceDataForFigure3(A)/A/Western Blots.pdf]

Figure 5C

Med.

*M. cat.*

WT

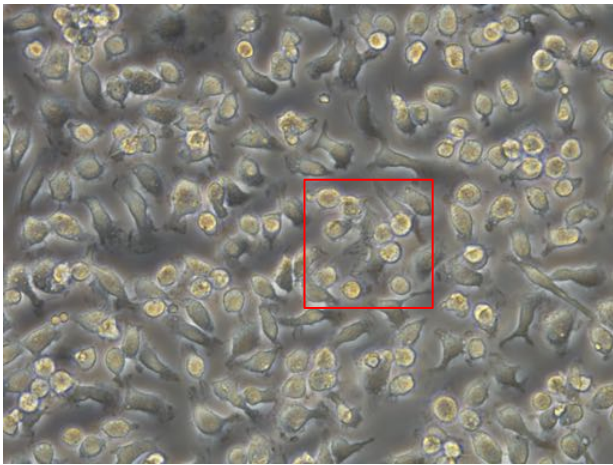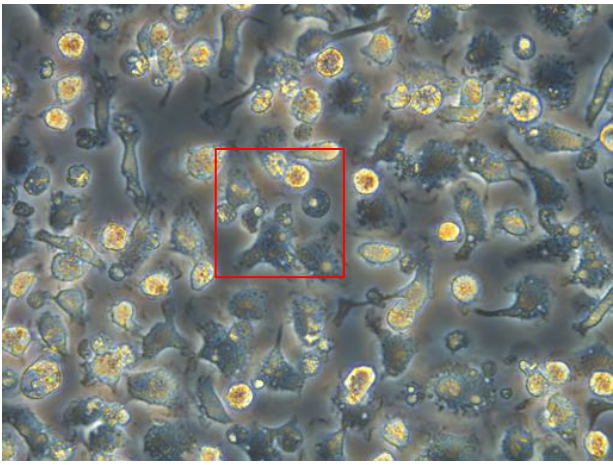

*Gbp1*<sup>-/-</sup>

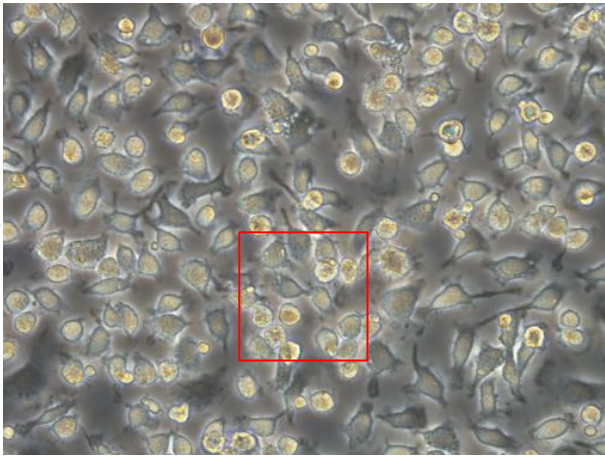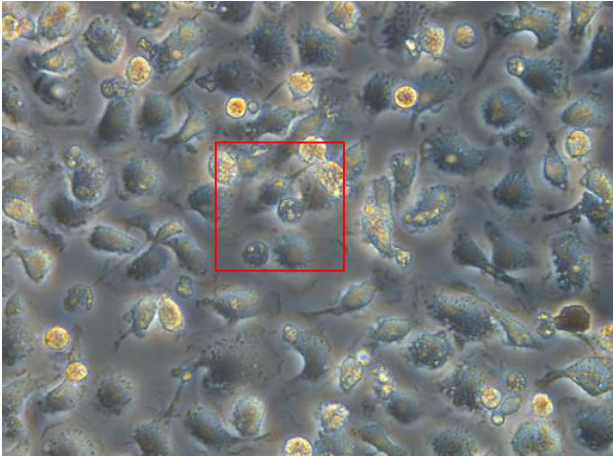

*Gbp2*<sup>-/-</sup>

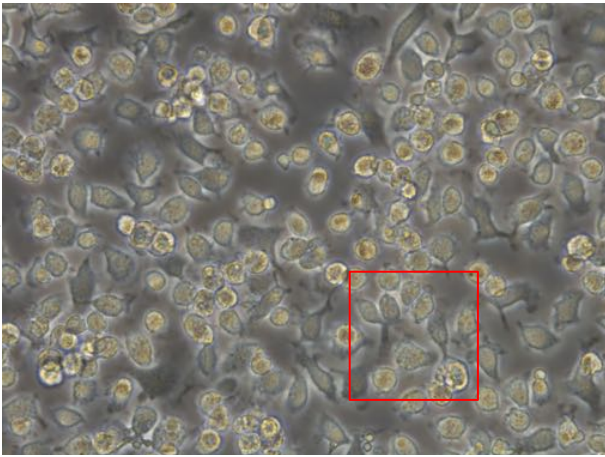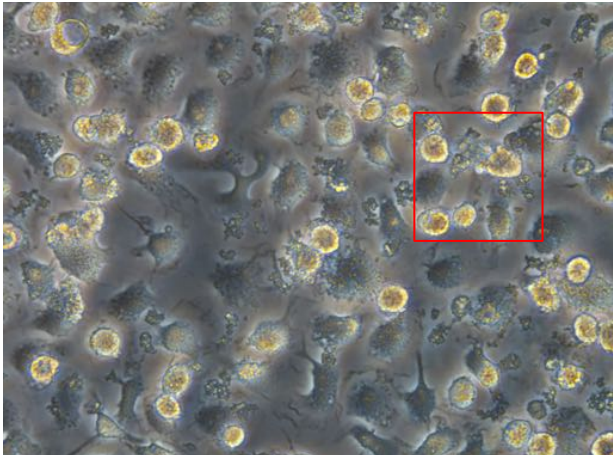

*Gbp3*<sup>-/-</sup>

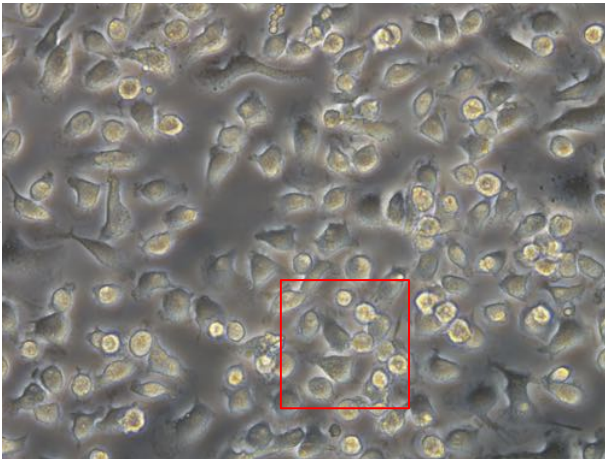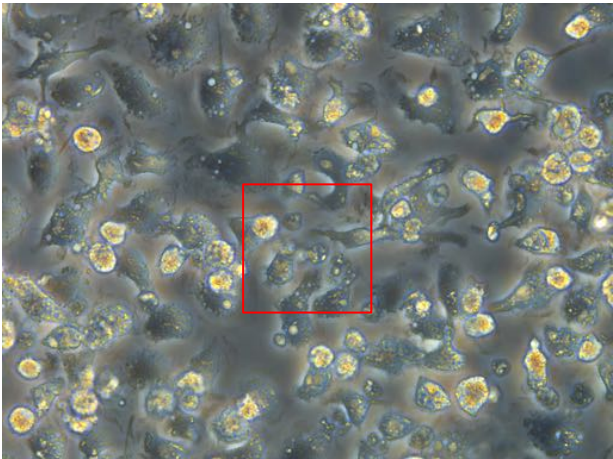

Figure 5C

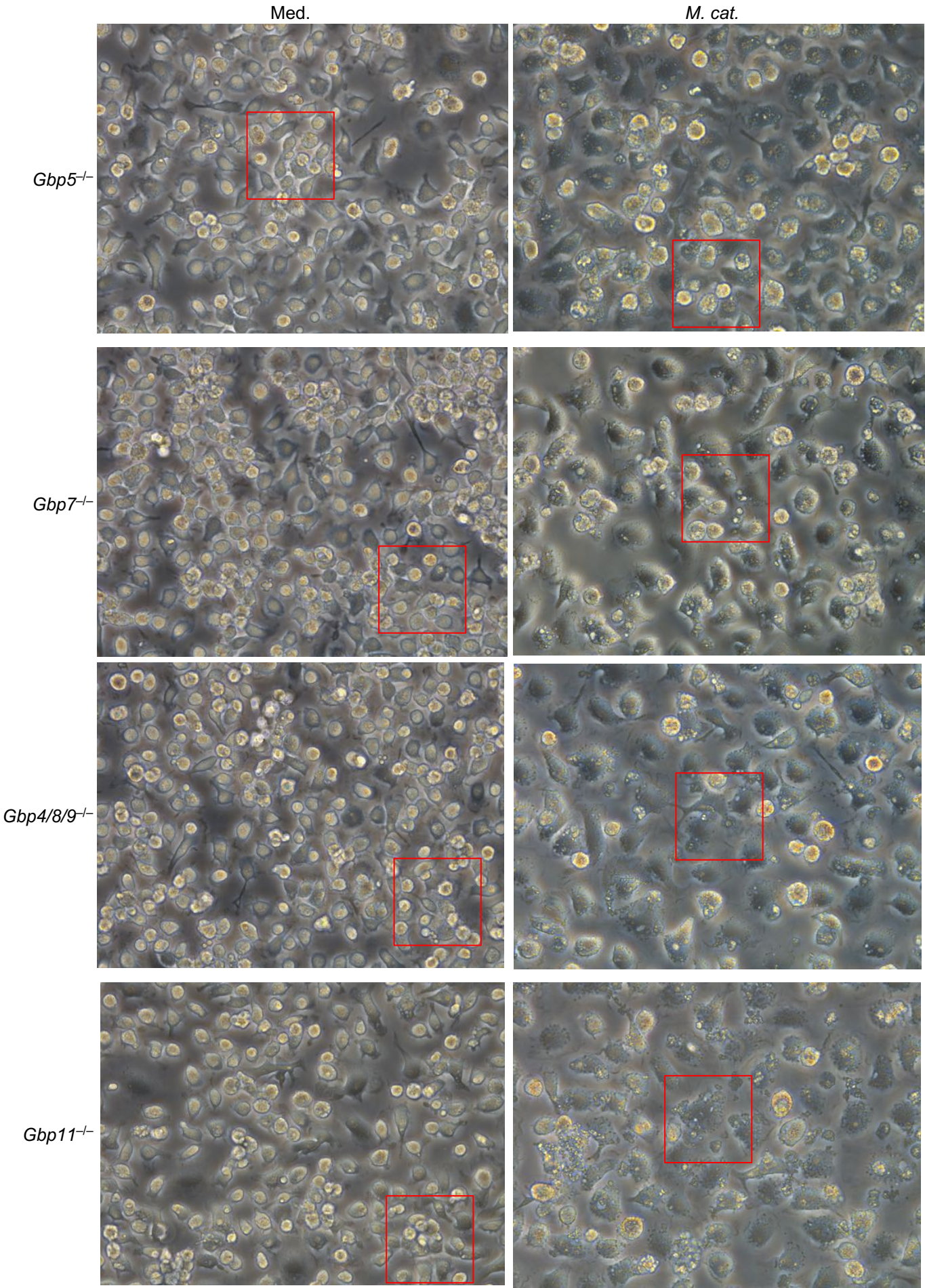

Figure 5C

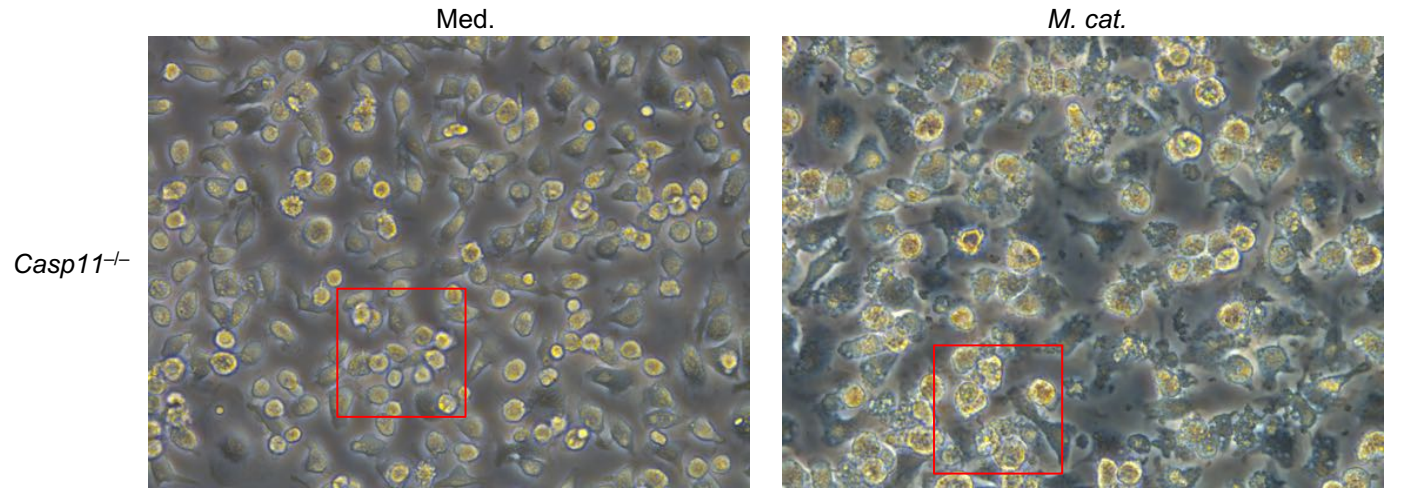

Supplement: Supplementary file 9 — Source Data for Figure 5 [file EMBJ-42-e112558-s004.zip › EMBOJ2022112558_SourceDataForFigure5(A,C)/C/Microscopy Image Bright-Field.pdf]

Figure 6A

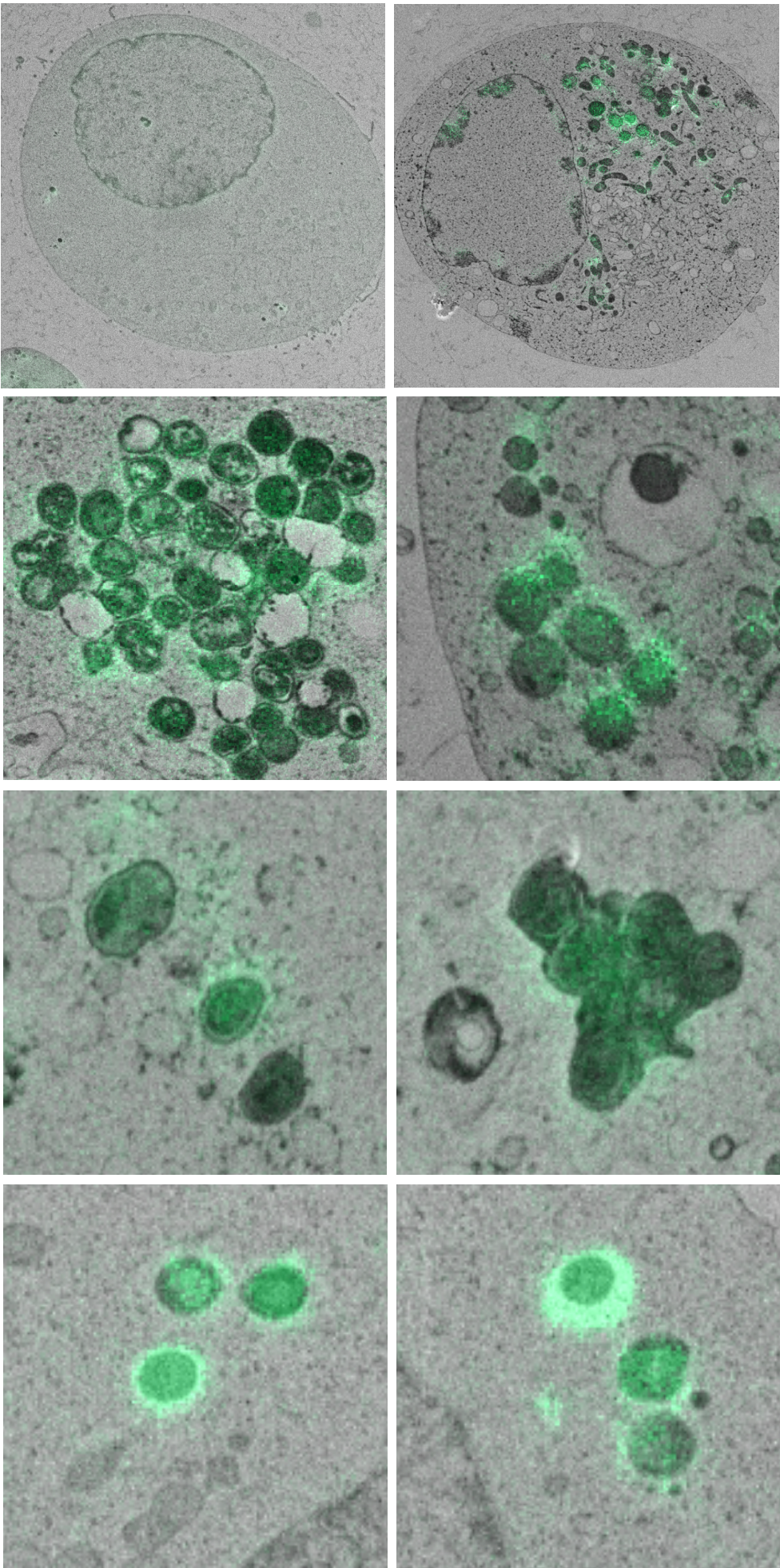

Supplement: Supplementary file 10 — Source Data for Figure 6 [file EMBJ-42-e112558-s005.zip › EMBOJ2022112558_SourceDataForFigure6(A,I,J)/A/Microscopy Image EM.pdf]

Figure 6I

Sol. Ctrl.

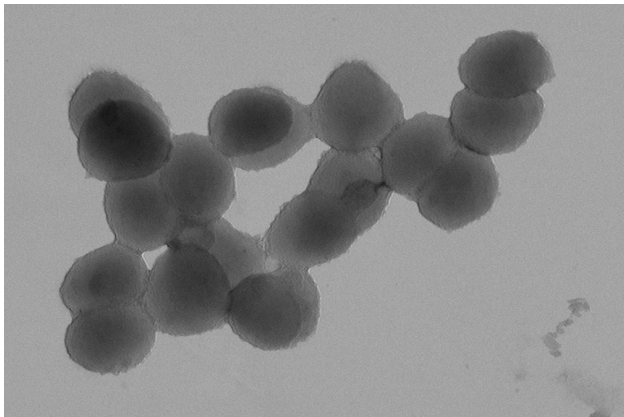

mGBP2

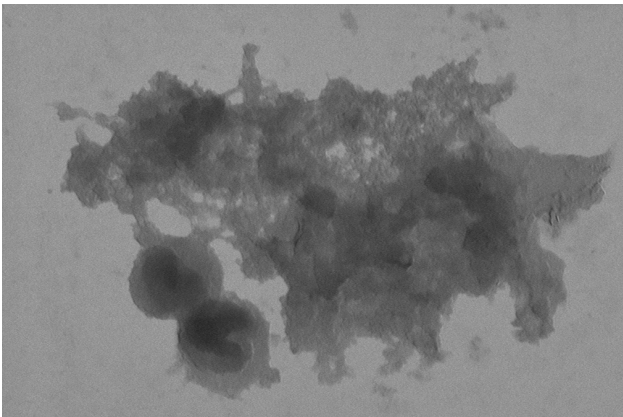

Supplement: Supplementary file 10 — Source Data for Figure 6 [file EMBJ-42-e112558-s005.zip › EMBOJ2022112558_SourceDataForFigure6(A,I,J)/I/Microscopy Image EM.pdf]

**Figure 6J**

Sol. Ctrl.

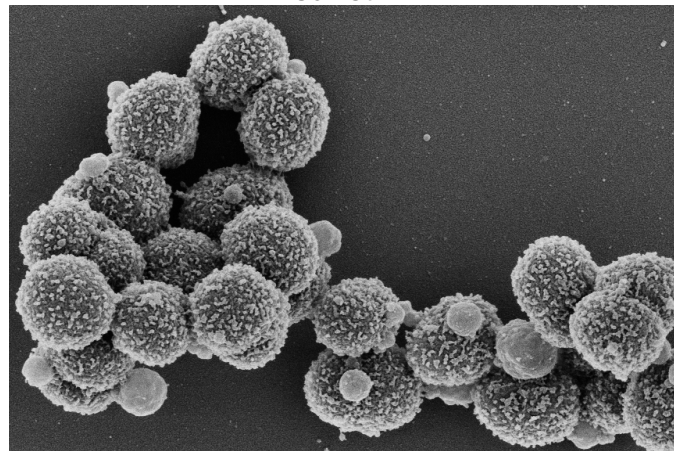

mGBP2

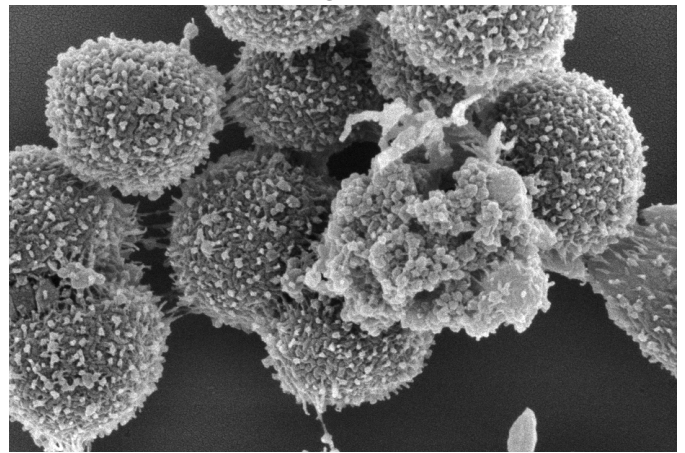

Supplement: Supplementary file 10 — Source Data for Figure 6 [file EMBJ-42-e112558-s005.zip › EMBOJ2022112558_SourceDataForFigure6(A,I,J)/J/Microscopy Image EM.pdf]
