# Supplementary material for: Immunity against Moraxella catarrhalis requires guanylate‐binding proteins and caspase‐11‐NLRP3 inflammasomes
Source: EMBO J. 2023 Feb 10;42(6):e112558. doi: 10.15252/embj.2022112558 (PMC10015372; doi:10.15252/embj.2022112558)

Figure 2C

- WT, *Nlrp3*$^{-/-}$, *Casp11*$^{-/-}$ BMDMs
- Media, *M. catarrhalis* (O35E) infection, *M. catarrhalis* infection (Δ*lpxA*)

Caspase-1

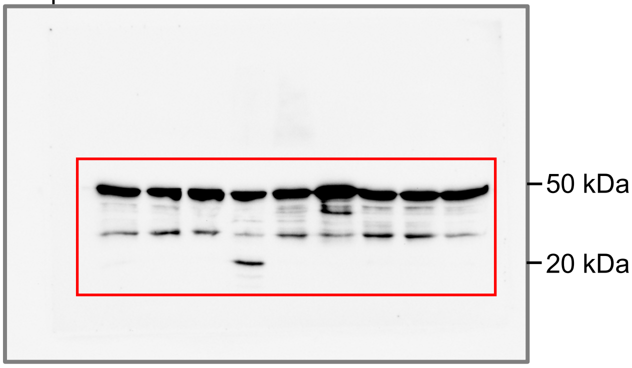

Caspase-11

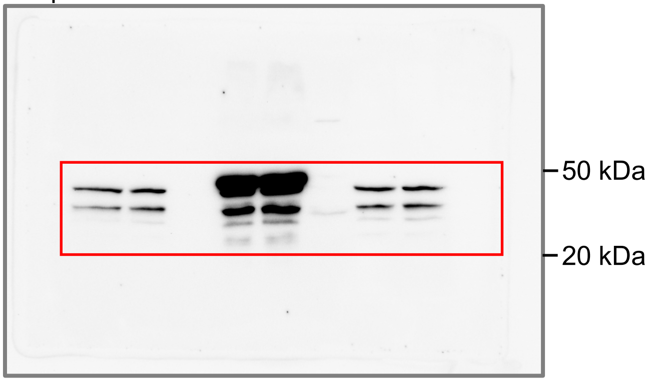

Gasdermin-D

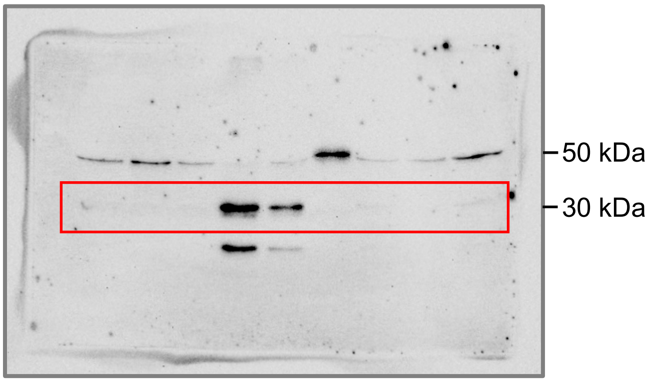

Supplement: Supplementary file 6 — Source Data for Figure 2 [file EMBJ-42-e112558-s002.zip › EMBOJ2022112558_SourceDataForFigure2(A,C,G,H,I)/C/Western Blots.pdf]
