# Supplementary material for: Immunity against Moraxella catarrhalis requires guanylate‐binding proteins and caspase‐11‐NLRP3 inflammasomes
Source: EMBO J. 2023 Feb 10;42(6):e112558. doi: 10.15252/embj.2022112558 (PMC10015372; doi:10.15252/embj.2022112558)

Figure 4C

- Media
- Merge/DAPI

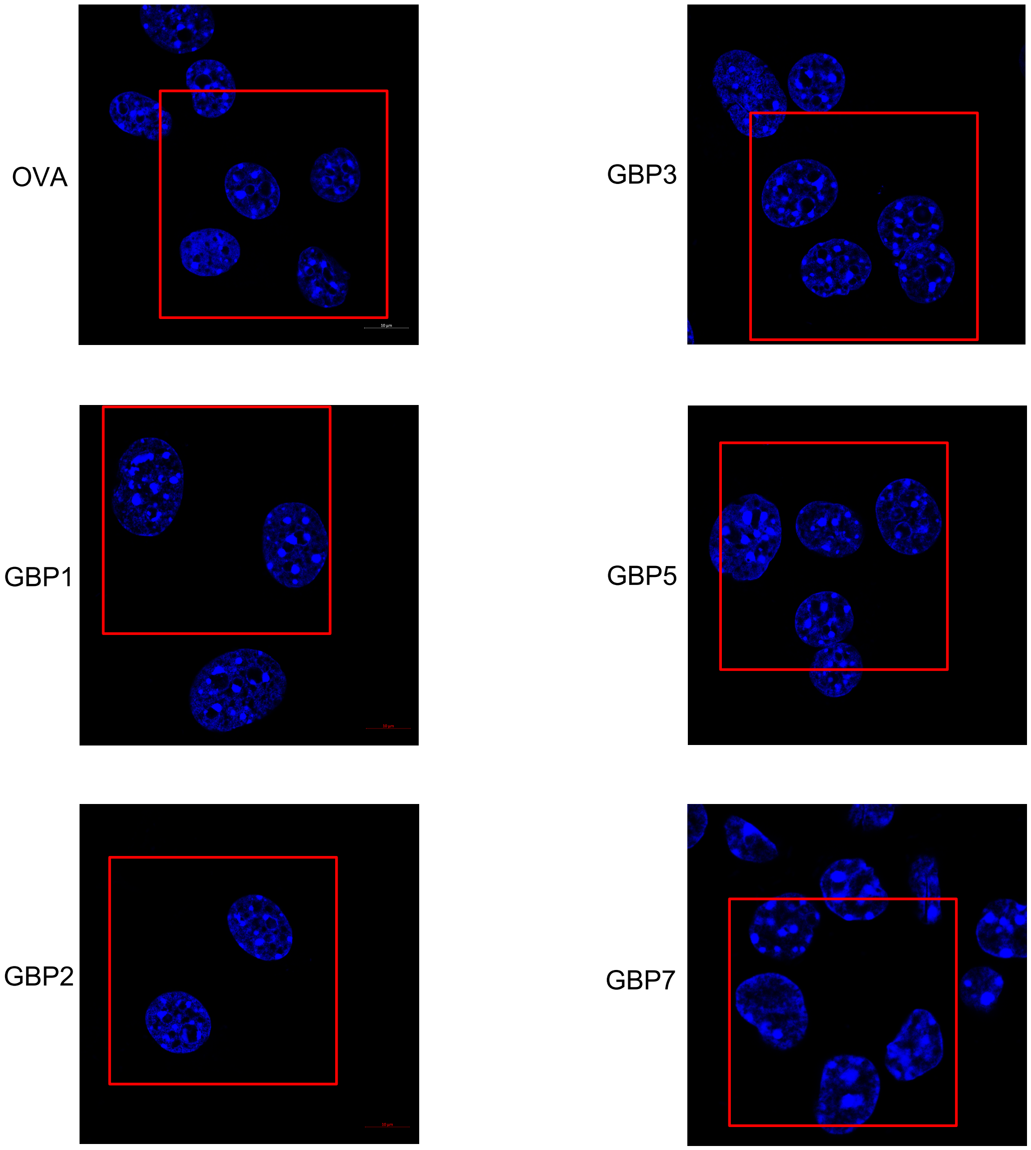

Figure 4C

- *M. catarrhalis* infection
- GBP (single channel)

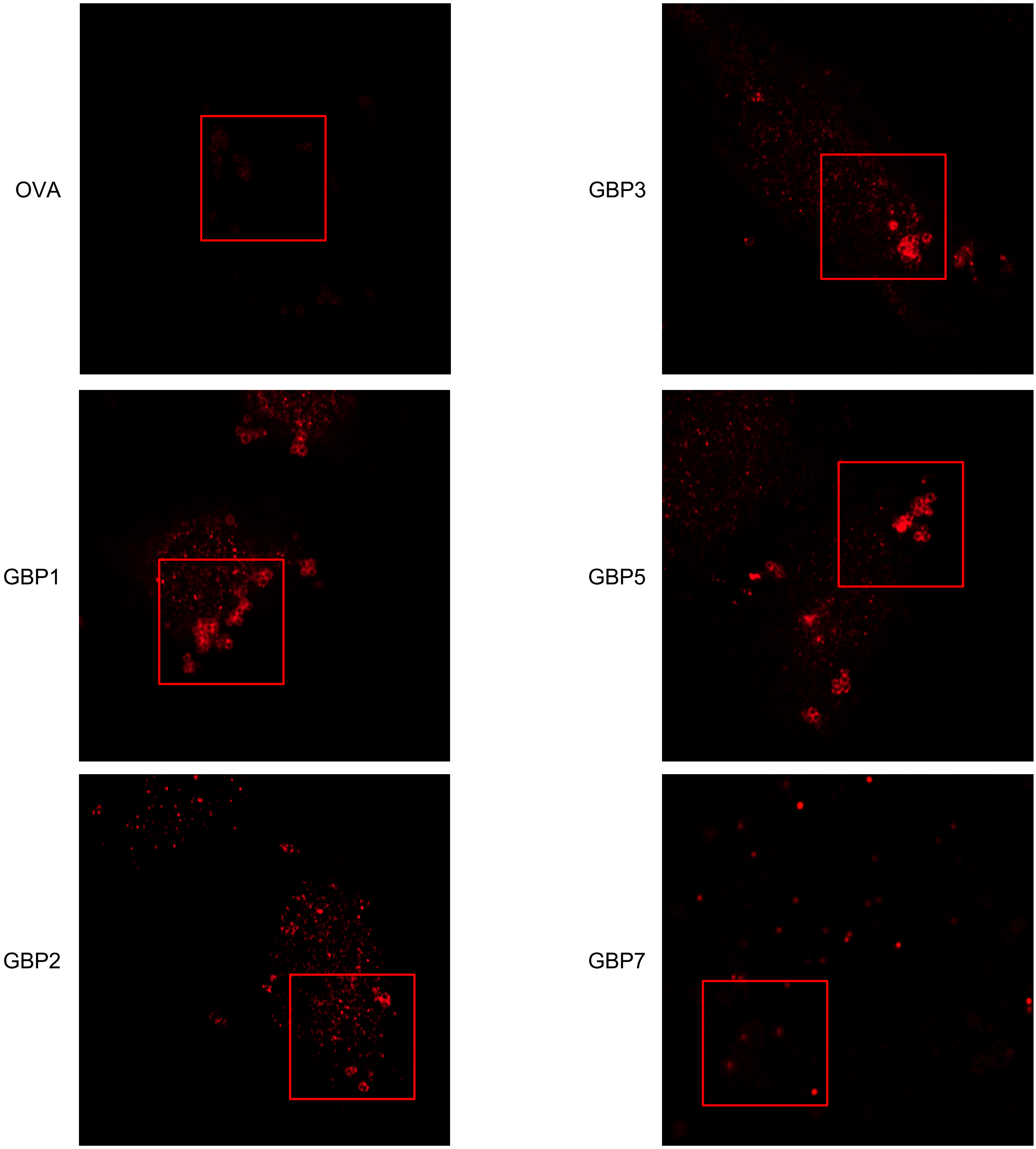

Figure 4C

- *M. catarrhalis* infection
- *M. cat.* (single channel)

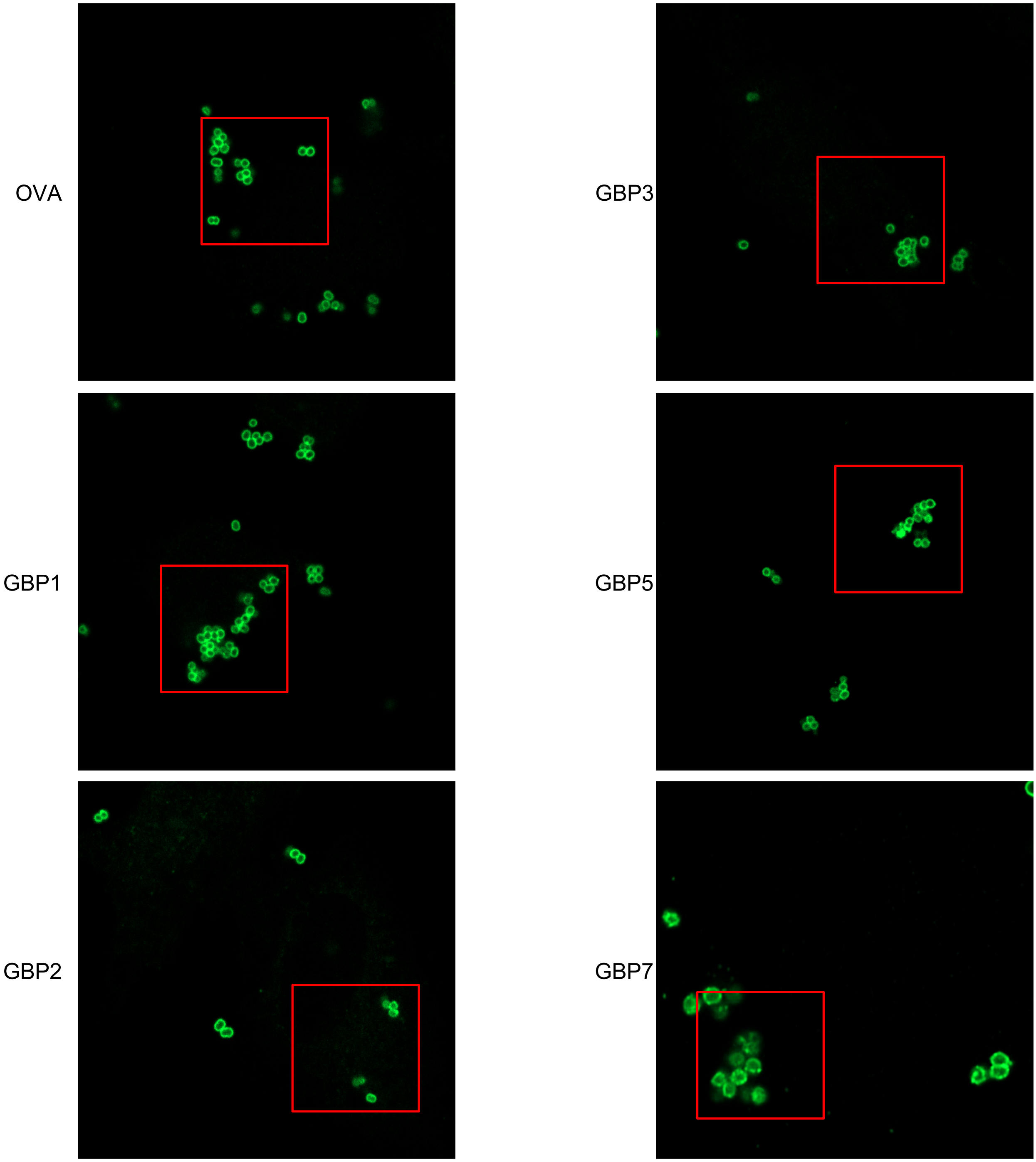

Figure 4C

- *M. catarrhalis* infection
- Merge/DAPI

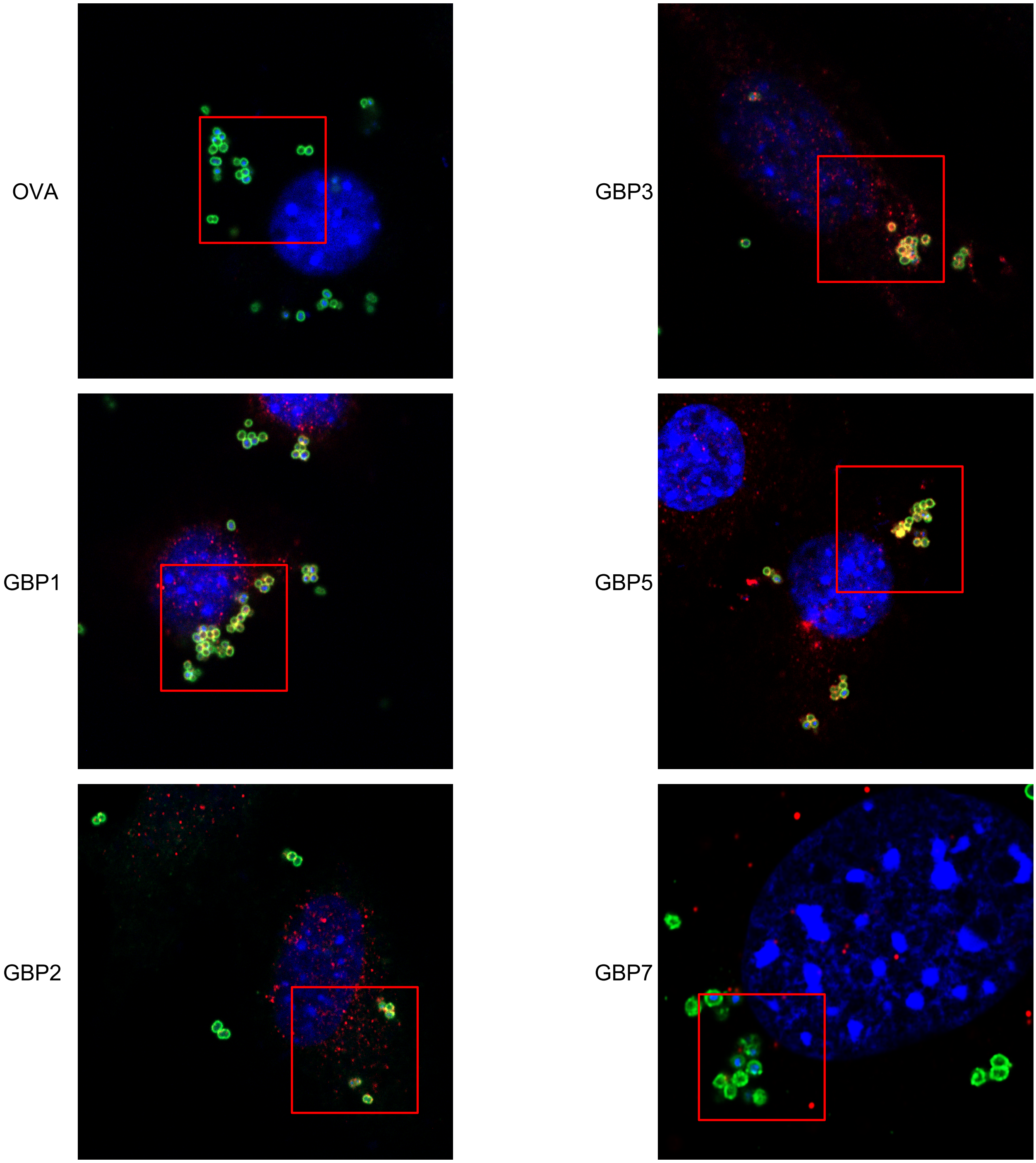

Supplement: Supplementary file 8 — Source Data for Figure 4 [file EMBJ-42-e112558-s010.zip › EMBOJ2022112558_SourceDataForFigure4(A,C,D)/C/Microscopy Image Confocal.pdf]
