# Supplementary material for: Immunity against Moraxella catarrhalis requires guanylate‐binding proteins and caspase‐11‐NLRP3 inflammasomes
Source: EMBO J. 2023 Feb 10;42(6):e112558. doi: 10.15252/embj.2022112558 (PMC10015372; doi:10.15252/embj.2022112558)

Figure 4D

- WT, *Gbp3*^{chr3}-KO, *Casp11*$^{-/-}$, BMDMs
- Media, *M. catarrhalis* infection, LPS transfection

Caspase-1 (*M. cat.* infection)

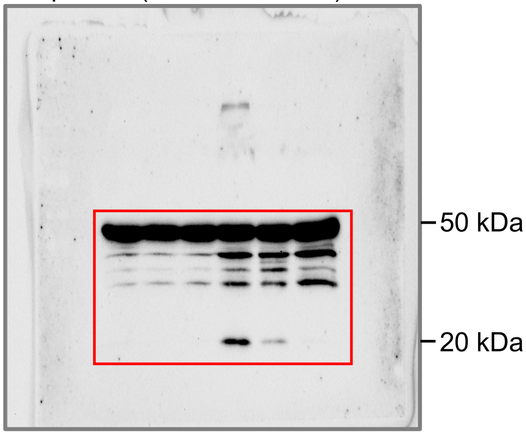

Caspase-1 (LPS transfection)

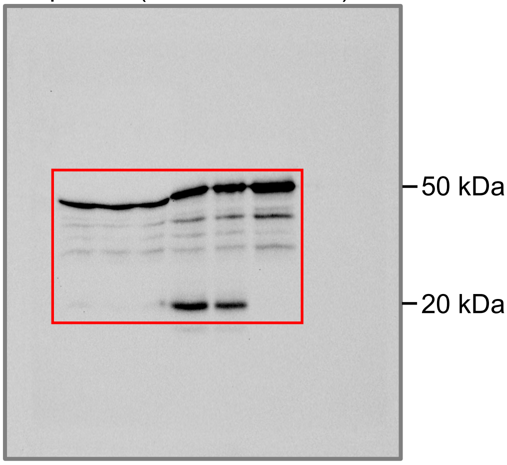

Caspase-11 (*M. cat.* infection)

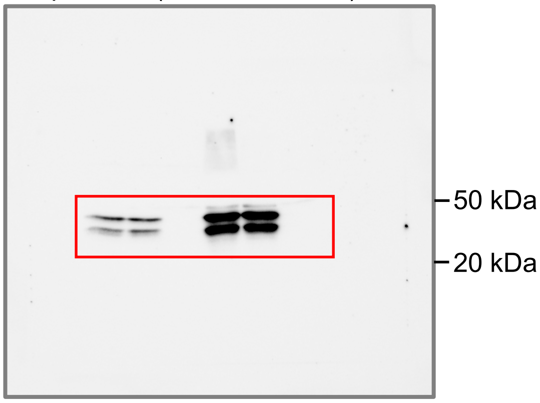

Caspase-11 (LPS transfection)

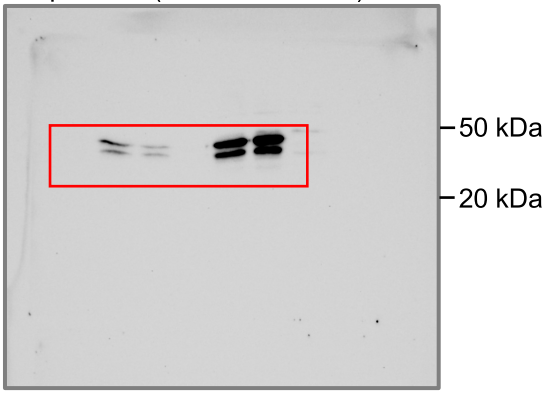

Gasdermin-D (*M. cat.* infection)

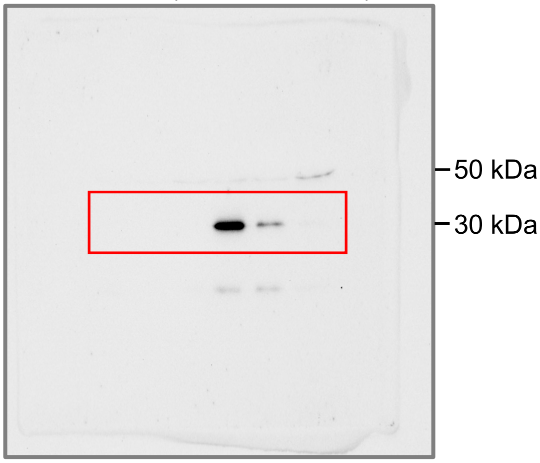

Gasdermin-D (LPS transfection)

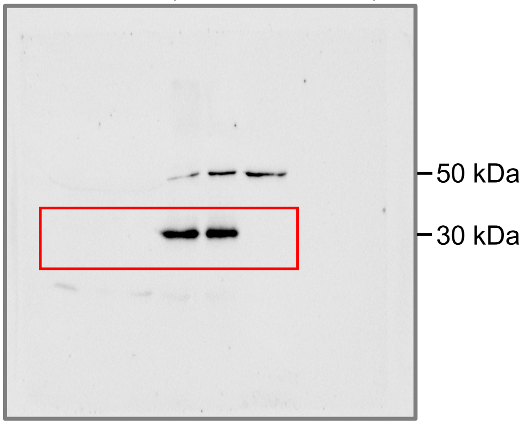

Supplement: Supplementary file 8 — Source Data for Figure 4 [file EMBJ-42-e112558-s010.zip › EMBOJ2022112558_SourceDataForFigure4(A,C,D)/D/Western Blots.pdf]
