# Supplementary material for: Immunity against Moraxella catarrhalis requires guanylate‐binding proteins and caspase‐11‐NLRP3 inflammasomes
Source: EMBO J. 2023 Feb 10;42(6):e112558. doi: 10.15252/embj.2022112558 (PMC10015372; doi:10.15252/embj.2022112558)

Figure 5A

- WT, *Gbp1*$^{-/-}$, *Gbp2*$^{-/-}$, *Gbp3*$^{-/-}$, *Gbp5*$^{-/-}$, *Gbp7*$^{-/-}$, *Casp11*$^{-/-}$ BMDMs
- Media, *M. catarrhalis* infection, LPS transfection

Caspase-1 (*M. cat.*)

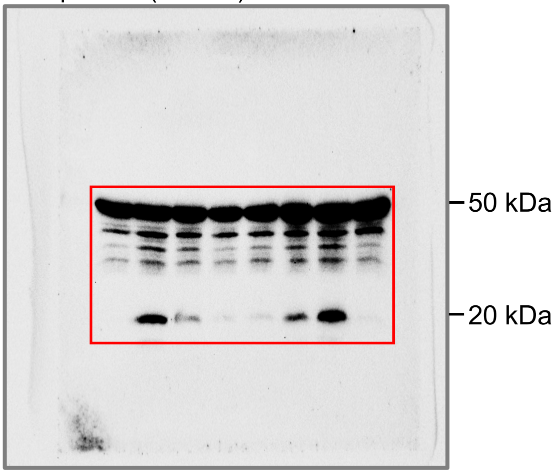

Caspase-1 (LPS trans.)

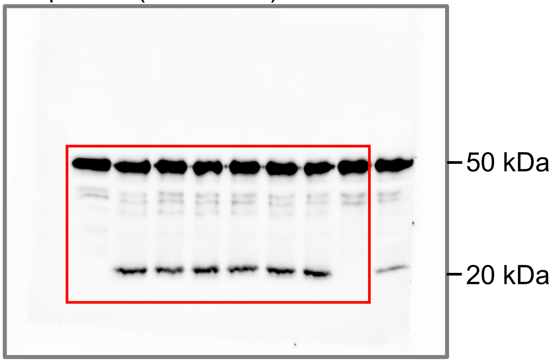

Caspase-11 (*M. cat.*)

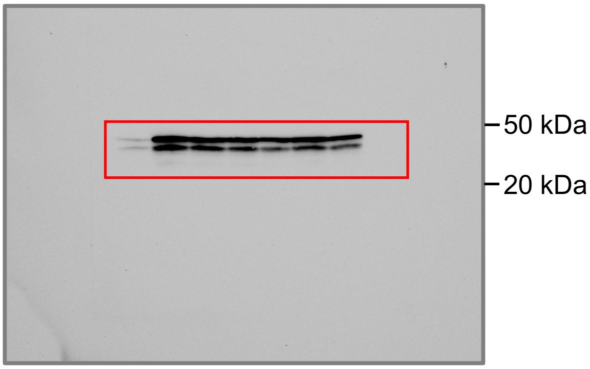

Caspase-11 (LPS trans.)

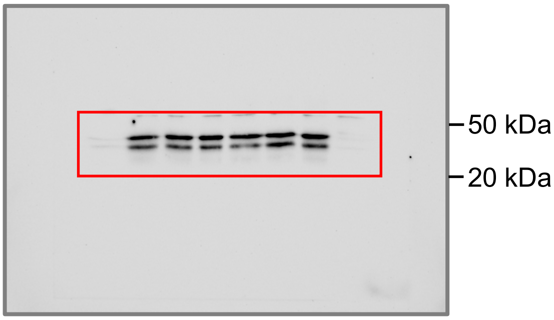

Gasdermin-D (*M. cat.*)

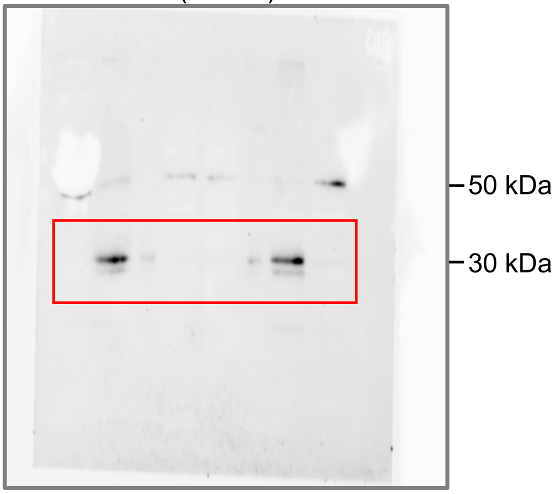

Gasdermin-D (LPS trans.)

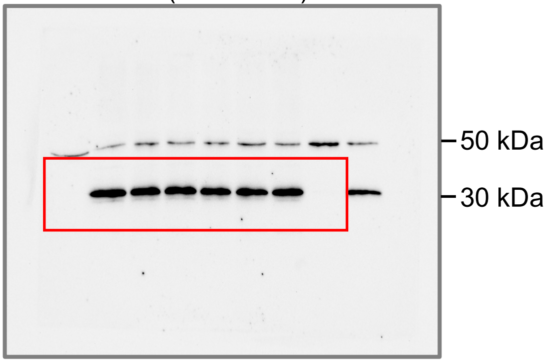

Figure 5A

- WT, *Gbp4/8/9*$^{-/-}$, *Gbp11*$^{-/-}$, *Casp11*$^{-/-}$ BMDMs
- Media, *M. catarrhalis* infection, LPS transfection

Caspase-1 (*M. cat.*)

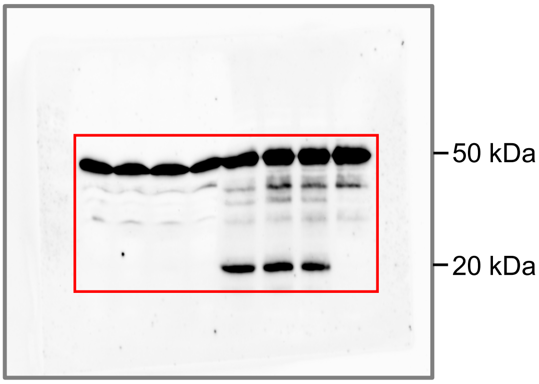

Caspase-1 (LPS trans.)

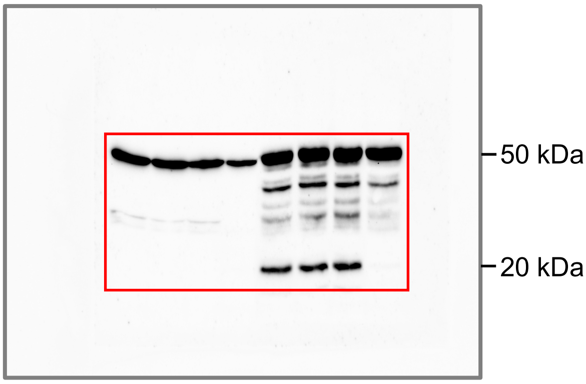

Caspase-11 (*M. cat.*)

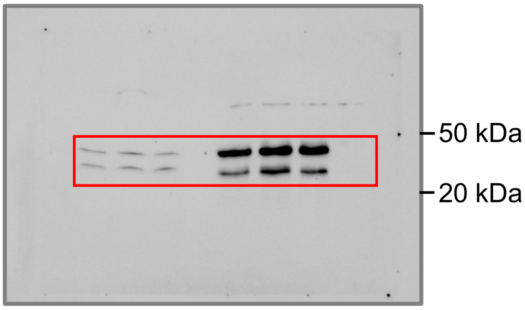

Caspase-11 (LPS trans.)

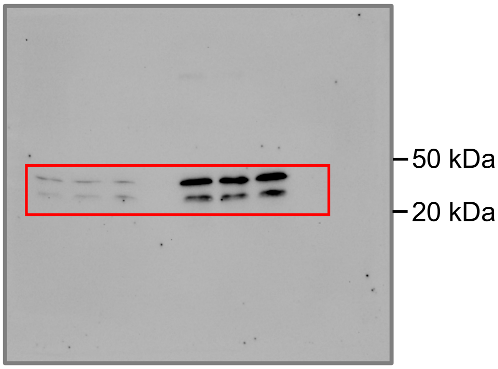

Gasdermin-D (*M. cat.*)

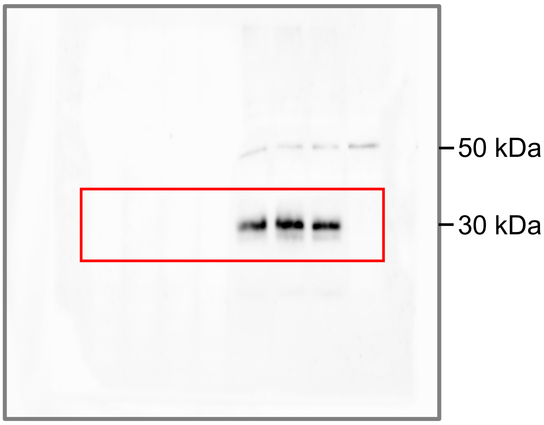

Gasdermin-D (LPS trans.)

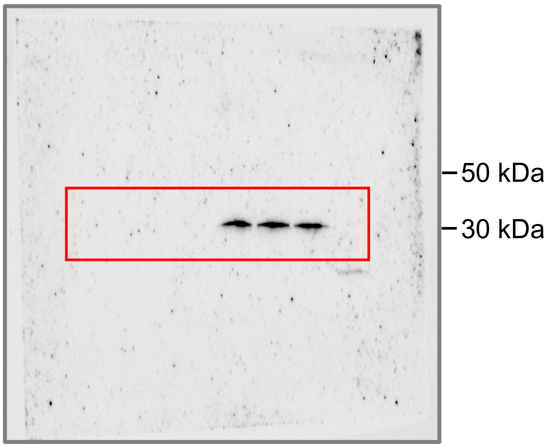

Supplement: Supplementary file 9 — Source Data for Figure 5 [file EMBJ-42-e112558-s004.zip › EMBOJ2022112558_SourceDataForFigure5(A,C)/A/Western Blots.pdf]
